# Dynamics and structural changes of calmodulin upon interaction with the antagonist calmidazolium

**DOI:** 10.1186/s12915-022-01381-5

**Published:** 2022-08-09

**Authors:** Corentin Léger, Irène Pitard, Mirko Sadi, Nicolas Carvalho, Sébastien Brier, Ariel Mechaly, Dorothée Raoux-Barbot, Maryline Davi, Sylviane Hoos, Patrick Weber, Patrice Vachette, Dominique Durand, Ahmed Haouz, J. Iñaki Guijarro, Daniel Ladant, Alexandre Chenal

**Affiliations:** 1 Biochemistry of Macromolecular Interactions Unit, Department of Structural Biology and Chemistry, CNRS UMR3528, Institut Pasteur, Paris, 75015 France; 2 Biological NMR and HDX-MS Technological Platform, CNRS UMR3528, Université Paris Cité, Institut Pasteur, Paris, 75015 France; 3 Université Paris Cité, Paris, France; 4 Plate-forme de Cristallographie-C2RT, Université Paris Cité, CNRS UMR3528, Institut Pasteur, Paris, France; 5 Plateforme de Biophysique Moléculaire, Université Paris Cité, CNRS UMR3528, Institut Pasteur, Paris, France; 6 Université Paris-Saclay, CEA, CNRS, Institute for Integrative Biology of the Cell (I2BC), 91198 Gif-sur-Yvette, France

**Keywords:** Calmodulin, CaM, Calmidazolium, CDZ, Calmodulin antagonist, Structure, Protein dynamics

## Abstract

**Background:**

Calmodulin (CaM) is an evolutionarily conserved eukaryotic multifunctional protein that functions as the major sensor of intracellular calcium signaling. Its calcium-modulated function regulates the activity of numerous effector proteins involved in a variety of physiological processes in diverse organs, from proliferation and apoptosis, to memory and immune responses. Due to the pleiotropic roles of CaM in normal and pathological cell functions, CaM antagonists are needed for fundamental studies as well as for potential therapeutic applications. Calmidazolium (CDZ) is a potent small molecule antagonist of CaM and one the most widely used inhibitors of CaM in cell biology. Yet, CDZ, as all other CaM antagonists described thus far, also affects additional cellular targets and its lack of selectivity hinders its application for dissecting calcium/CaM signaling. A better understanding of CaM:CDZ interaction is key to design analogs with improved selectivity. Here, we report a molecular characterization of CaM:CDZ complexes using an integrative structural biology approach combining SEC-SAXS, X-ray crystallography, HDX-MS, and NMR.

**Results:**

We provide evidence that binding of a single molecule of CDZ induces an open-to-closed conformational reorientation of the two domains of CaM and results in a strong stabilization of its structural elements associated with a reduction of protein dynamics over a large time range. These CDZ-triggered CaM changes mimic those induced by CaM-binding peptides derived from physiological protein targets, despite their distinct chemical natures. CaM residues in close contact with CDZ and involved in the stabilization of the CaM:CDZ complex have been identified.

**Conclusion:**

Our results provide molecular insights into CDZ-induced dynamics and structural changes of CaM leading to its inhibition and open the way to the rational design of more selective CaM antagonists.

**Graphical abstract:**

Calmidazolium is a potent and widely used inhibitor of calmodulin, a major mediator of calcium-signaling in eukaryotic cells. Structural characterization of calmidazolium-binding to calmodulin reveals that it triggers open-to-closed conformational changes similar to those induced by calmodulin-binding peptides derived from enzyme targets. These results provide molecular insights into CDZ-induced dynamics and structural changes of CaM leading to its inhibition and open the way to the rational design of more selective CaM antagonists.
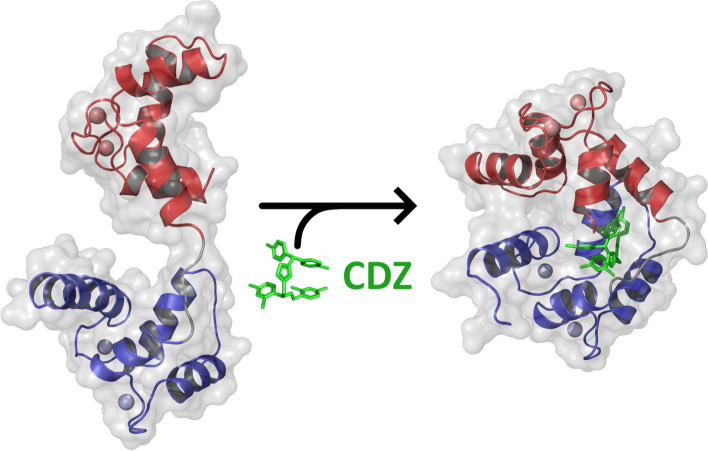

**Supplementary Information:**

The online version contains supplementary material available at 10.1186/s12915-022-01381-5.

## Background

Calmodulin (CaM) is an essential 148 amino acid-long calcium binding protein that is ubiquitously found in all eukaryotes and one of the most conserved proteins known to date, playing a central role in cell physiology as a key sensor of intracellular calcium signaling [1–3]. It regulates a wide variety of biochemical processes by interacting in a calcium-dependent manner with numerous effector proteins to modulate their enzymatic activities and/or their structural properties. Calmodulin has four calcium binding sites, called EF-hand motifs, made of about 30 amino acids that adopt a helix-loop-helix fold [4]. Calcium binds with high affinity to the loop flanked by the two helical segments. CaM has two pairs of EF-hands (N-lobe and C-lobe) connected by a flexible linker. Calcium binding to EF-hands triggers large conformational changes that result in the exposure of hydrophobic patches and promote interaction with target proteins [5, 6]. Structural studies have highlighted the remarkable plasticity of CaM upon association with its effectors. In many instances, CaM binds its target proteins *via* short, 20–30 residues long, CaM-binding sites (CBS) with a positively charged, amphipathic alpha-helical character. Calcium-bound CaM (holo-CaM) generally binds by wrapping its two lobes around the CBS helix [7], adopting widely diverse configurations that allow it to match the great variability of the primary amino-acid sequences of CBS [1, 8–10]. For many effectors, the CBS sequence is located close to an auto-inhibitory domain that maintains the target enzyme in an inhibited state until holo-CaM binding to the CBS relieves the inhibition [7, 10]. Alternatively, holo-CaM can interact with targets in a noncanonical fashion in which the N- and C-lobes can simultaneously and independently associate to either distinct sites on the same protein or to the same site on two distinct polypeptide chains, triggering oligomerization [7, 11, 12]. Besides, CaM can also associate with various proteins regardless of the presence of calcium, frequently *via* an “IQ motif” that interacts with both calcium-bound and calcium-free CaM (Apo-CaM) [7, 13].

CaM mediates many physiological processes such as inflammation, metabolism, proliferation, apoptosis, smooth muscle contraction, intracellular movement, short-term and long-term memory, and immune responses [2, 10, 12, 14, 15]. Given its central role in cell physiology, molecules able to antagonize CaM activity are in dire need for fundamental studies of calcium/CaM signaling as well as for potential therapeutic applications. A variety of compounds with diverse chemical structures were characterized in the 80s following the initial observations that the phenothiazine family of antipsychotic drugs could inhibit the actions of holo-CaM [16–21]. Many pharmacologically active drugs (antidepressants, anticholinergics, smooth muscle relaxants, local anesthetics, antiparasitic) were also shown to bind holo-CaM. It was suggested that their therapeutic activity might be, in part, related to their ability to interfere with the calcium/CaM signaling axis, which was therefore seen as a potentially relevant pharmacological target [21, 22]. At a fundamental level, these drugs have also been widely used to probe the role of CaM in cell biology. A major issue with these CaM antagonists is their lack of selectivity as most of them also target multiple additional effectors, making difficult to ascribe specific effects to their calcium/CaM signaling pathway inhibition [23, 24]. Although these CaM antagonists display a variety of chemical structures, they generally share overall hydrophobicity and a net positive charge. Their CaM-binding affinities as well as their stoichiometries are also variable. Structures of several of these antagonists in complex with CaM have revealed the diversity in their protein binding mode, akin to the variability of CaM-CBS complexes [25–30].

Here we provide a detailed characterization of the structural and dynamic changes of holo-CaM induced by the binding of calmidazolium (CDZ), one of the most potent and widely used CaM antagonists [19, 22, 31–34]. We demonstrate both in crystal and in-solution that the binding of a single CDZ, primarily to the N-lobe of holo-CaM, is enough to induce an open-to-closed conformational reorientation of the two domains of CaM associated with drastic changes of CaM dynamics. This is in marked contrast to prior studies that speculated that multiple CDZ could bind a single CaM molecule. We further delineate the structural rearrangements and changes in internal dynamics triggered by CDZ binding to CaM, as well as the dynamics of the association. These structural data will be instrumental to design CDZ analogs with improved selectivity for CaM.

## Results

### CDZ binding to holo-CaM monitored by SRCD and ITC

We first investigated the effect of CDZ on the secondary structure content of CaM using circular dichroism in the far-UV range to determine whether CDZ binding impacts CaM folding (Fig. S1). The addition of CDZ does not induce major changes of the far-UV CD spectrum of calcium-loaded calmodulin (holo-CaM), suggesting that CDZ binding does not significantly alter the secondary structure content of holo-CaM. We then investigated the thermodynamics of CDZ binding to holo-CaM by isothermal titration calorimetry (ITC). The data indicated an apparent equilibrium dissociation K_D_ constant of 3 ± 2 μM with a stoichiometry of 1.2 ± 0.5 (Fig. S2), in agreement with Dagher et al. [33]. An integrative structural biology approach combining size-exclusion chromatography coupled to small-angle X-ray scattering (SEC-SAXS), X-ray crystallography (XR), hydrogen deuterium exchange—mass spectrometry (HDX-MS), and nuclear magnetic resonance (NMR) was then used to provide molecular insights into the CDZ-induced dynamics and conformational changes of holo-CaM, leading to its inhibition [19, 22, 31–35].

### Analysis of CDZ-induced holo-CaM conformational changes by SEC-SAXS

We analyzed the effects of CDZ binding on the molecular shape of holo-CaM by SEC-SAXS measurements [36]. The experiments were performed in the presence of 5% DMSO to prevent CDZ aggregation (see Supplementary information for details). The SEC-SAXS patterns recorded for holo-CaM in the absence and in the presence of 5% DMSO were essentially identical, suggesting that 5% DMSO has no detectable effects on holo-CaM (Fig. S3).

**Fig. 1:**
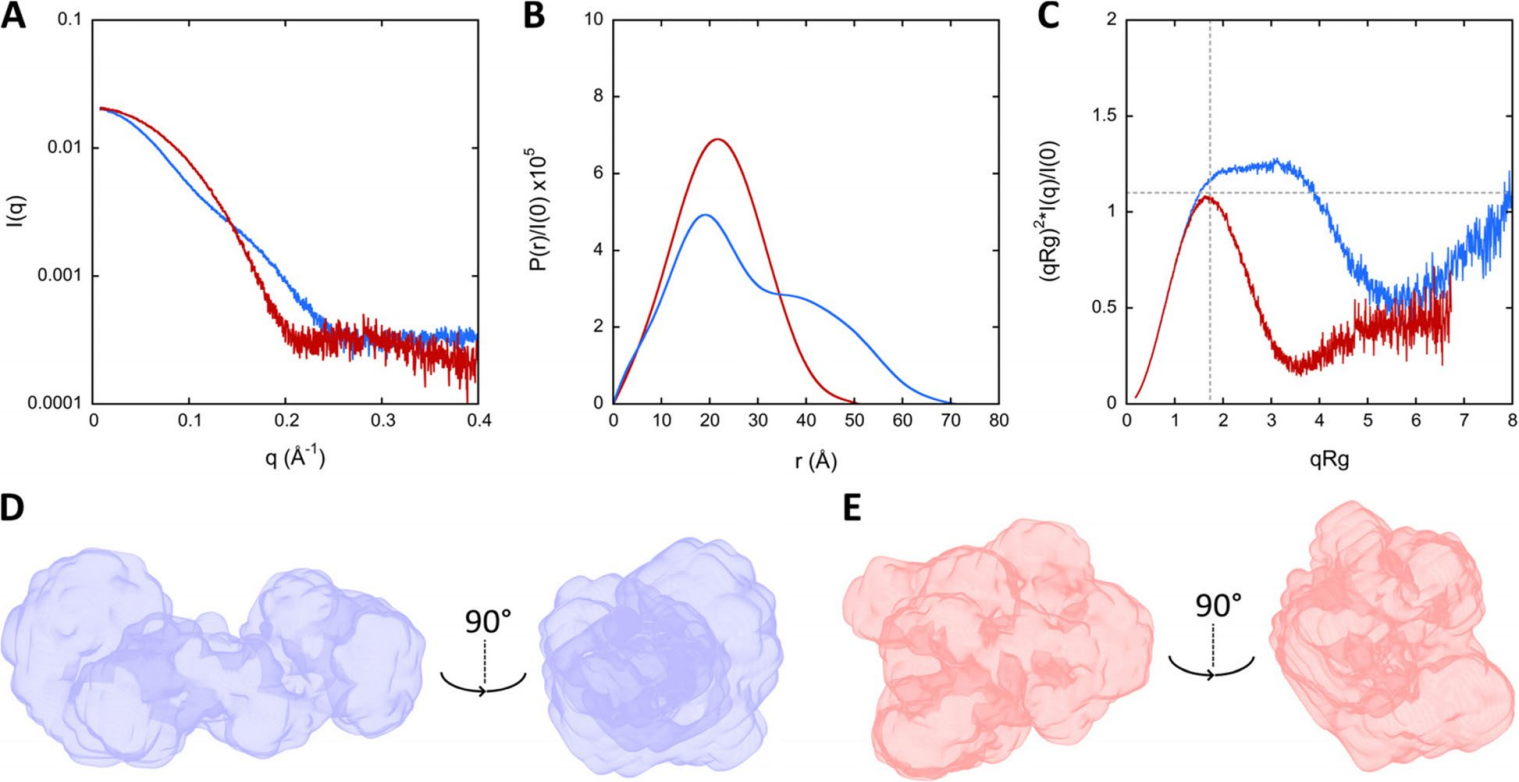
SAXS patterns and DENSS analysis of holo-CaM in the absence and in the presence of CDZ. **A** Scattering patterns of holo-CaM (blue trace) and holo-CaM:CDZ complex (red trace). **B** Distance distribution function P(r) of holo-CaM in the absence and in the presence of CDZ using GNOM, same color code as in panel **A**. **C** Dimensionless Kratky representation of holo-CaM in the absence and in the presence of CDZ, same color code as in panel **A**. Dash lines crossing *x*-axis (√3) and *y*-axis (1.104) indicate the characteristic peak for globular proteins. **D, E** DENSS models of holo-CaM and holo-CaM:CDZ, respectively

The SEC-SAXS patterns of holo-CaM in the absence and in the presence of CDZ are shown in Fig. 1A and the derived structural parameters are reported in Table S1. The SAXS patterns exhibit dramatic differences that are further highlighted by the pair-distance distribution function, P(r), and the dimensionless Kratky analysis (Fig. 1B, C). The distance distribution functions show that holo-CaM adopts a bi-lobed conformation (SASBDB ID: SASDNX3) and that CDZ binding induces a compaction of the protein, leading to a globular shape (SASBDB ID: SASDNY3). The CDZ-induced conformational change of holo-CaM is characterized by a dramatic reduction of the radius of gyration, R_g_, from 22.4 to 16.8 Å and of the maximal interatomic distances, D_max_, from 72 Å to 52 Å (Fig. 1B and Table S1). The dimensionless Kratky plots indicate a significant reduction of the structural flexibility [36] and maximal dimension of holo-CaM upon CDZ binding (Fig. 1C). Indeed, the holo-CaM:CDZ complex exhibits the archetypical profile of a folded, compact, and isometric protein while the broad peak shifted to higher coordinate values reveals an extended conformation of holo-CaM implying mutual mobility of the two lobes around the flexible central linker. Ab initio models of CaM global conformations were calculated using DENSS [37], yielding a bi-lobed extended shape for holo-CaM and a globular shape for the holo-CaM:CDZ complex (Fig. 1D, E), in agreement with the pair-distance distribution functions and the dimensionless Kratky plots (Fig. 1B, C). Taken together, the SAXS results indicate that CDZ-binding induces an open-to-closed conformational reorientation of the two domains of CaM and a reduction of holo-CaM flexibility, illustrating the conformational plasticity of holo-CaM.

### Structural analysis of holo-CaM by SAXS and X-ray crystallography

**Fig. 2:**
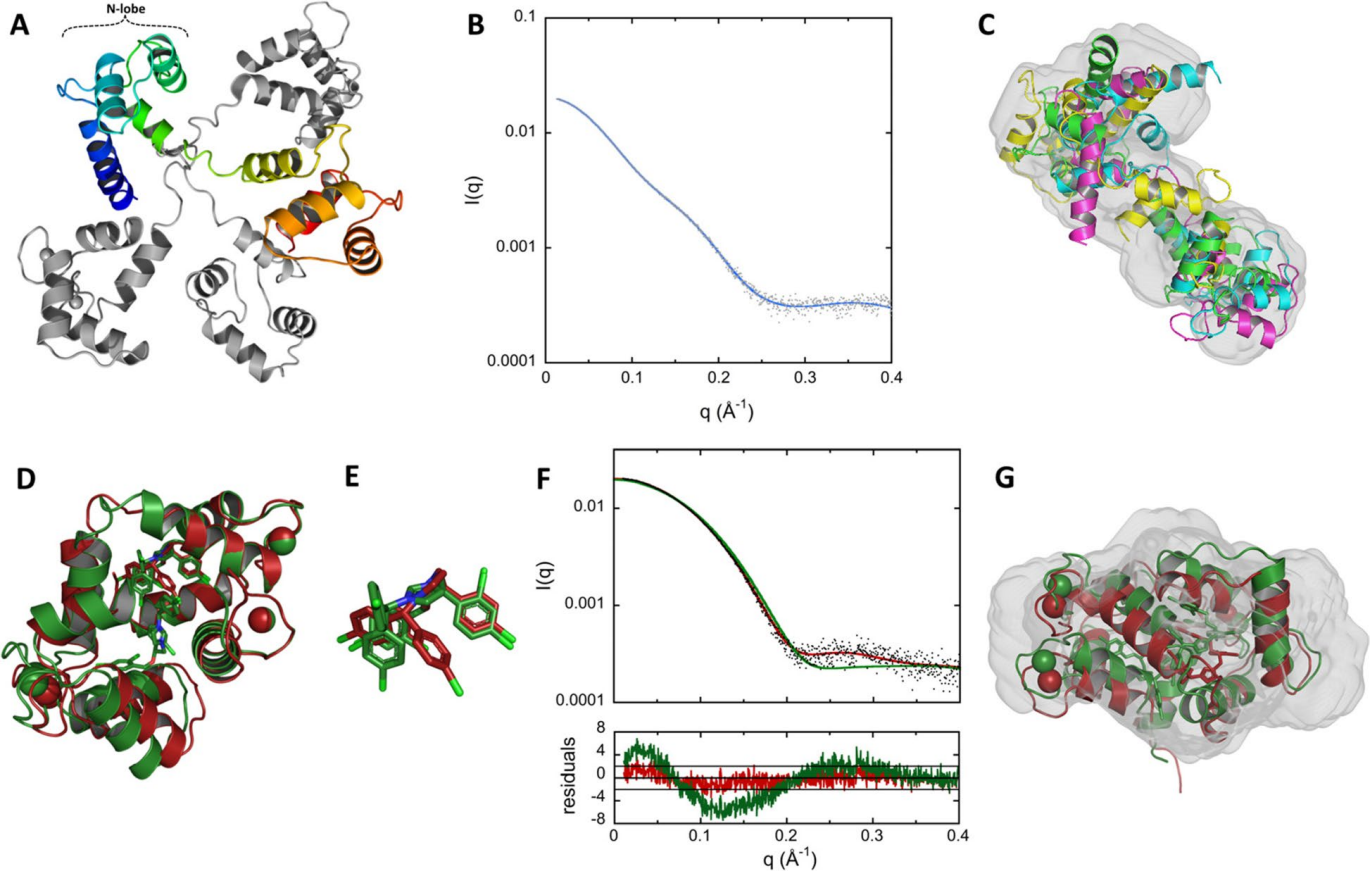
Structural models of holo-CaM using EOM and structures of holo-CaM:CDZ determined by X-ray crystallography. **A** Final ensemble of SAXS-derived conformations of holo-CaM using EOM. The four structural models (SASBDB ID: SASDNX3) are superimposed by aligning their N-lobe alpha carbons (residues 6 to 66) using Pymol. One structural model is shown in rainbow, the three others are in grey. **B** Comparison of experimental data (grey dots) to the calculated scattering pattern (blue curve) of the final EOM ensemble. **C** Fitting of the four EOM structural models of holo-CaM to the SAXS-derived DENSS volume. **D** X-ray structures of holo-CaM in complex with one (red, PDB ID: 7PSZ) and two (green, PDB ID: 7PU9) CDZ molecules. **E** Superimposition of the CDZ molecules of both structures showing the rotation of the chlorophenyl moieties. **F** Top: Fitting of the calculated scattering patterns of the two crystallographic structures of holo-CaM:CDZ obtained using Crysol to the experimental SAXS pattern recorded with 333 μM of CaM and 1050 μM of CDZ. The *χ*^2^ are 1.4 and 9.3 for the 1:1 (red) and 1:2 (green) holo-CaM:CDZ complexes, respectively. Bottom: Distribution of reduced residuals corresponding to the two fits presented above**. G** Fitting of the two crystallographic structures of holo-CaM:CDZ to the SAXS-derived DENSS volume (SASBDB ID: SASDNY3)

The Ensemble Optimization Methods (EOM) suite of programs [38, 39] was used to generate an ensemble of structural models of holo-CaM. Briefly, ten thousand conformations were generated by the Ranch program, starting from the structures of the two calcium-bound lobes of holo-CaM (PDB ID: 1CLL [40]). Residues (76–81) in the interlobe linker region were described as dummy residues. These residues were then substituted by a full-atom description with PD2 [41] and the energy of each linker was minimized using SCWRL4 [42]. The scattering pattern of each of the 10,000 conformations was then computed using CRYSOL [43]. Finally, the Gajoe program was used to select ensembles of conformation, the average scattering pattern of each ensemble being fitted against the experimental data using a genetic algorithm. The best ensemble for holo-CaM (SASBDB ID: SASDNX3) displays the typical conformation of holo-CaM with two well-folded lobes connected by a highly flexible linker (Fig. 2A) and shows a good fit to the experimental scattering pattern (Fig. 2B) with a *χ*^2^ value of 1.066. The four models in this ensemble fit quite well inside the DENSS [37] volume (Fig. 2C).

The holo-CaM:CDZ complex was investigated using X-ray crystallography. Depending on the molar ratio of holo-CaM and CDZ, we obtained two crystal forms. For the holo-CaM:CDZ complex prepared at a molar ratio of 1:2.2 (1 mM holo-CaM), the unit-cell parameters, merging statistics, and systematic absences were consistent with space group C121, while for the holo-CaM:CDZ complex prepared at a molar ratio of 1:10 (1 mM holo-CaM), the parameters were coherent with space group P6_1_22. The corresponding structures PDB ID 7PSZ (holo-CaM:CDZ_A_ for holo-CaM with one CDZ molecule, CDZ-A) and PDB ID 7PU9 (holo-CaM:CDZ_BC_ for holo-CaM with two CDZ molecules, CDZ-B and CDZ-C) were solved by molecular replacement to 1.9 Å and 2.3 Å, respectively (Fig. 2D and Table S2). In the complex prepared at a 1:2.2 molar ratio of holo-CaM:CDZ (PDB ID: 7PSZ), some density was left unattributed inside holo-CaM. This density, which could be assigned to a second CDZ (Fig. S4), may arise from partial occupation due to differences between molecules within the unit-cell or random sub-stoichiometric binding.

The two crystal structures of holo-CaM:CDZ with one and two CDZ molecules are quite similar with a RMSD of only 1.4 Å over all Cα and exhibit compact and globular structures (Fig. 2D). CDZ-A and CDZ-B are bound to the same region of holo-CaM and are mostly localized in the N-lobe. The superimposition of the N-lobes of the two crystal structures shows that the conformation of CDZ-A and CDZ-B slightly differ by the rotation of two chlorophenyl groups (Fig. 2E). This rotation seems required to accommodate the second CDZ (CDZ-C) inside the holo-CaM:CDZ_BC_ complex. Analysis with LigPlot+ [44] indicates that holo-CaM:CDZ interactions are mostly driven by hydrophobic effects (Fig. S5), with CDZ-A and CDZ-B mainly interacting with residues from the C-terminal part of the N-lobe and the N-terminal part of the C-lobe of holo-CaM, as shown in Table S3. These residues are similar to those identified by Reid et al. [45]. CDZ-C mainly interacts with the N- and C-terminal extremities of CaM.

The crystal structures and the SEC-SAXS data of holo-CaM:CDZ were compared using DENSS and Crysol. Both crystal structures fit well within the envelope of the DENSS model generated with the SEC-SAXS data of the holo-CaM:CDZ complex (Fig. 2G). Comparison of the theoretical SAXS patterns of the two crystal structures generated by Crysol to the experimental SEC-SAXS pattern of the complex indicates that the holo-CaM:CDZ_A_ crystal structure is in excellent agreement with experimental data with a *χ*^2^ of 1.4, against 9.3 for the holo-CaM:CDZ_BC_ crystal structure (Fig. 2F). Taken together, these analyses indicate that the holo-CaM:CDZ complex exhibits a similar conformation in solution and in the crystals and suggest that a single CDZ molecule is sufficient to induce an open-to-closed conformational reorientation of the two domains of holo-CaM. These results suggest that the part of CDZ_A_, which remains solvent-exposed once interacting with the N-lobe of holo-CaM, likely forces the C-lobe to collapse by a hydrophobic effect, closing the two lobes of holo-CaM on themselves, with holo-CaM wrapping around CDZ_A_.

### HDX-MS analysis of holo-CaM upon CDZ binding

**Fig. 3:**
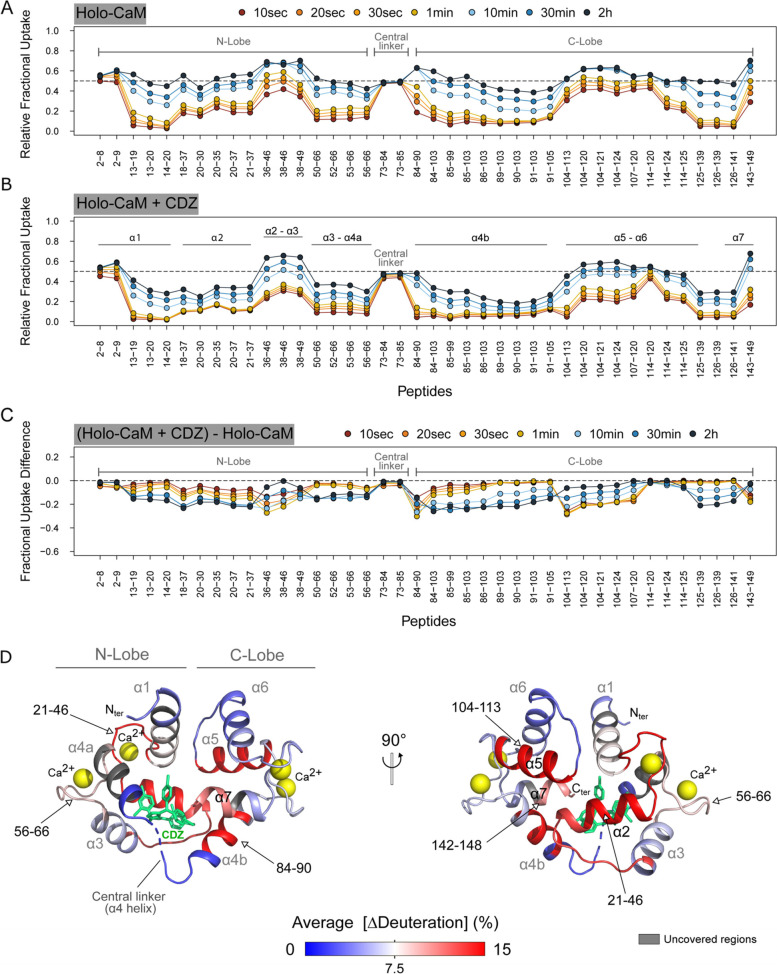
Effects of CDZ binding on the deuterium uptake profile of holo-CaM. **A**, **B** Relative fractional uptake plots of holo-CaM measured in the presence and in the absence of 20 μM CDZ. Each dot corresponds to the average uptake value measured in three independent replicates. **C** The effects of CDZ binding on holo-CaM are visualized on the fractional uptake difference plot. Negative values indicate a reduction in solvent accessibility induced by CDZ binding. **D** Cartoon representation of holo-CaM showing the average differences in “fractional uptake differences” between the CDZ-bound and free holo-CaM states. The fractional uptake differences ([ΔDeuteration] in %) measured between the CDZ-bound and free states were extracted for each peptide at each labelling time point, averaged, and plotted on the crystal structure of CaM:CDZ_A_ (PDB ID: 7PSZ). CDZ-A is colored in green. The average ΔDeuteration values [Average (ΔDeuteration)] are colored from blue (no variation) to red (major reductions in uptake)

The effect of CDZ binding on the solvent accessibility of holo-CaM was also investigated by HDX-MS. The holo-CaM:CDZ complex used in HDX-MS was prepared at a 1:32 molar ratio (0.63 μM CaM and 20 μM CDZ) in the presence of 2% DMSO (Table S4, Figs. S6 and S7). The excess of CDZ ensures that most of holo-CaM (circa 90 %) remains complexed during labeling (i.e., using a K_D_ of 3 ± 2μM and a 1.2 binding stoichiometry). CDZ binding induces similar reductions in solvent accessibility within the N- and C-lobes of holo-CaM (Fig. 3A–C) and has no effect on regions covering residues 2–9 (N-ter), 73–84 (interlobe linker region), and 114–124 (helix α6).

The effects of CDZ are best illustrated and visualized in Fig. 3D. This figure was generated by plotting on the CaM:CDZ_A_ crystal structure (PDB ID: 7PSZ, Fig. 2D) the average “Differences in Uptake differences” calculated between CDZ-bound and free holo-CaM for all labelling time points (Fig. S7). The main reductions in solvent accessibility are located in region 21–45 (calcium binding loop 1, α2) of the N-lobe, and in regions 84–90 (α4b), 104–113 (α5), and 143–149 (α7) of the C-lobe.

The regions with high reductions in solvent accessibility identified by HDX-MS compare well with the residues interacting with CDZ-A and CDZ-B in the crystal structures, as highlighted with Ligplot+ (Table S3). The residues interacting with CDZ-C in the crystal structure experience only a weak reduction in solvent accessibility, suggesting that CDZ-C is essentially absent in holo-CaM:CDZ complexes at the concentrations used for HDX-MS experiments. Finally, a weak reduction in solvent accessibility is measured for the interlobe linker region. The HDX-MS results appear therefore consistent with the crystal structure of the holo-CaM:CDZ_A_ complex and confirm that CDZ binding to the N-lobe dramatically stabilizes both lobes.

The effects of CDZ binding on the exchange behavior of holo-CaM were finally compared to those induced by the binding of CaM-binding peptides: two peptides, the H-helix and P454 derived from the adenylate cyclase toxin CyaA from *Bordetella pertussis* [46–49] and MLCK peptides [7, 35, 49]. The interlobe linker region (peptide 73-84) remains accessible to the solvent in the absence and in the presence of ligands (Fig. S8). Interestingly, CDZ, the MLCK, and the H-helix peptides induce similar differences in HDX uptake of holo-CaM, albeit with distinct amplitudes but the global patterns remain very similar. These results indicate that CDZ mimics the effects of biological ligands on holo-CaM. Taken together, HDX-MS data shows that CDZ binding to holo-CaM dramatically reduces the solvent accessibility of both lobes without affecting the interlobe linker region.

### Effect of CDZ binding on holo-CaM hydrodynamic parameters monitored by NMR

We evaluated the influence of CDZ binding to holo-CaM on the tumbling correlation time (τ_c_) at 37 °C with increasing equivalents of CDZ (Table S5). The *τ*_c_ value were determined using a ^15^N cross-correlated spin relaxation experiment to avoid possible contributions from chemical exchange (binding/unbinding) in the intermediate regime (μs–ms time scale). The observed increase in τ_c_ indicates that while the N- and C-lobes of holo-CaM tumble independently of each other (*τ*_c_ = 4.5 ± 0.2 ns, in agreement with previously published results [50]), the two lobes tumble together in a more compact holo-CaM:CDZ 1:1 complex (5.2 ± 0.3 ns). The *τ*_c_ value of CDZ-bound holo-CaM agrees well with the value calculated for a globular, compact protein of molecular mass and partial specific volume corresponding to holo-CaM (5.2 ns), further confirming the SAXS and X-ray analyses.

### Effects of CDZ on holo-CaM NMR spectra and chemical shifts

**Fig. 4:**
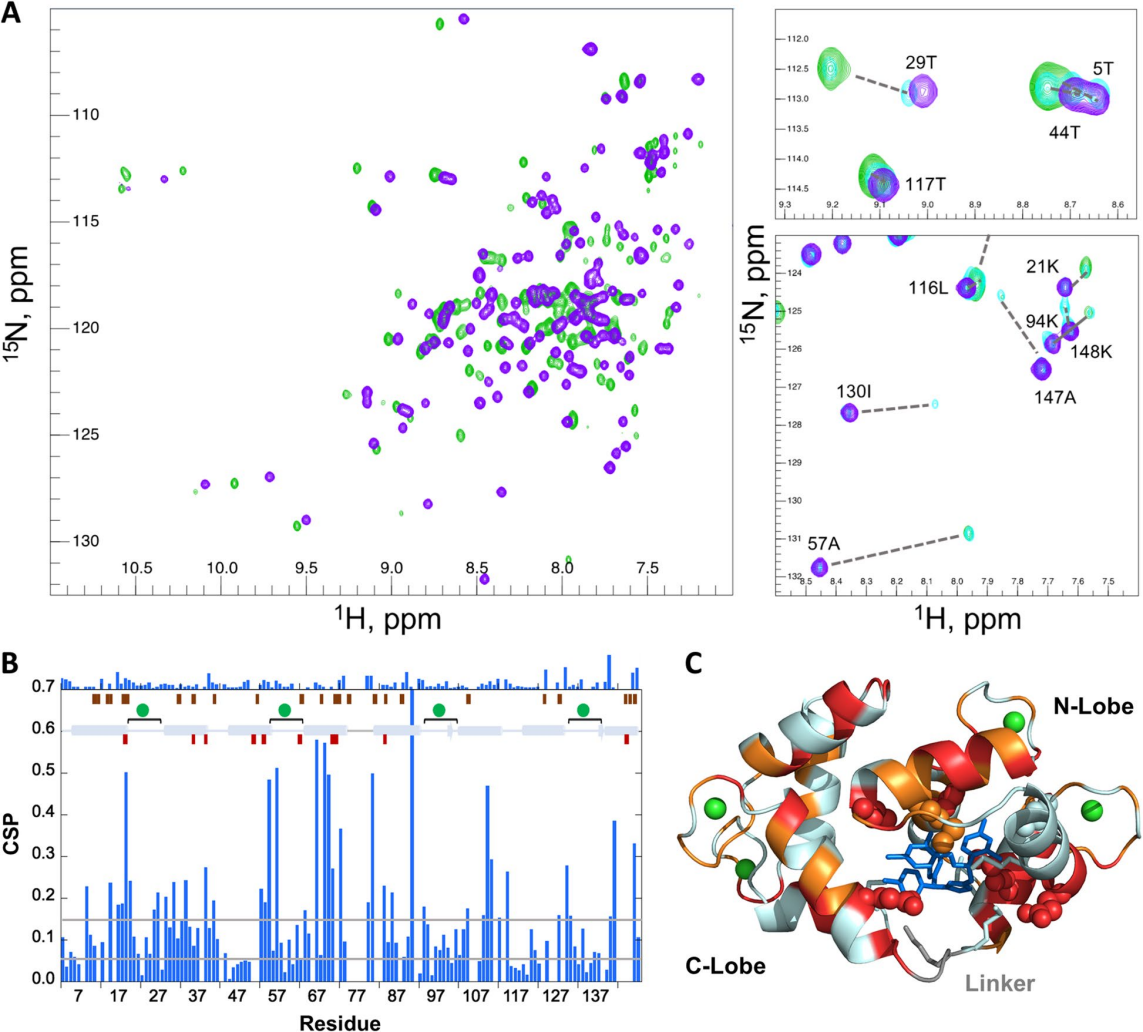
CDZ binding monitored by CaM ^1^H-^15^N chemical shift perturbation (CSP). **A** (left) ^1^H-^15^N SOFAST full fingerprint spectra (recorded at 37 °C) of holo-CaM alone (mauve) and in the presence of 1.0 equivalent of CDZ (green). (right) Zoom on two selected regions of the fingerprint spectra. The spectrum of holo-CaM in the presence of 0.5 equivalents (cyan) is also displayed. The assignments of free holo-CaM are shown and the dotted lines indicate the corresponding signal in the 1:1 holo-CaM:CDZ sample. **B** CSP values of 1 CDZ equivalent added to holo-CaM as a function of the residue number. The secondary structure (helix=cylinder, strand=arrow), calcium binding loops (spheres and square brackets), and linker region (grey line) are schematized. The CSP values between the 1:1 and 1:2 holo-CaM:CDZ complexes are displayed on the top of the panel, respecting the same scale. The positions of contacting residues in the X-ray 1:1 and 1:2 holo-CaM:CDZ complex structures are represented by wine and maroon rectangles, respectively. Grey lines represent the CSP values chosen as thresholds for very strong and strong CSPs. **C** Residues with very strongly perturbed (red, CSP ≥ 0.14) and strongly perturbed (orange, 0.07 ≤ CSP < 0.14) amide resonances are highlighted on the cartoon representation of the 1:1 holo-CaM:CDZ x-ray complex structure. CDZ is shown as blue sticks and Ca^2+^ ions as green spheres. The side chains of the CaM residues in close contact with CDZ as defined by Ligplot+ are highlighted as spheres if assigned (19, 36, 39, 54, 63, 71, 84) or as sticks (51, 72, 76, 77, and 145) if not observed by NMR. Amide resonances of the following residues were not assigned in the holo-CaM:CDZ complexes: 8, 12, 14, 16, 38, 51–52, 72, 75–79, 82–83, 88, 92, 106–107, 112, 114, 124, 126–127, 129–130, 139, 143-146

We recorded ^1^H-^15^N correlation spectra of holo-CaM in the absence and in the presence of 0.5 and 1 equivalents of CDZ (Fig. 4A). CDZ binding dramatically perturbs most holo-CaM signals, indicating that CDZ affects not only the signals of residues at the binding interface but also exhibits long-range effects on the conformations and/or internal motions throughout most of the protein. The spectrum of holo-CaM with a half-equivalent of CDZ (cyan, Fig. 4A, right panels) corresponds to the sum of the spectra of the free and the 1:1 molar ratio of holo-CaM:CDZ complex. Thus, (i) the free and bound holo-CaM conformations are in slow exchange on the chemical shift time scale as expected for a high affinity interaction and (ii) at sub-stoichiometric concentrations, one CDZ molecule binds to one holo-CaM. Binding of CDZ also affects the relative intensities of the ^1^H-^15^N signals and hence the internal motions of holo-CaM (see 130I signal in Fig. 4A).

As evaluated from the backbone and beta carbon (CB) resonances of the free and CDZ-bound holo-CaM using Talos-N, CDZ binding did not alter the secondary structure of holo-CaM, which was consistent with the secondary structure of the crystal structures determined herein and of holo-CaM in solution [51] (Table S6). Hence, the variations of chemical shifts in ^1^H-^15^N correlation spectra were due to residues in contact with CDZ, and/or to a modification of the tertiary structure and/or the internal dynamics of the protein.

We then performed a quantitative analysis of the chemical shift perturbations (CSPs) of holo-CaM upon binding to CDZ (Fig. 4B, C). Most of the highly affected residues were localized in regions contacting CDZ. Some strong perturbations were also located far away from the binding interface, for instance in calcium binding loops. Overall, the CSPs are in agreement with the binding interface observed in the holo-CaM:CDZ_A_ complex (Fig. 4C) and further indicate that CDZ binding affects distal regions in holo-CaM. Importantly, of the 31 unassigned amide resonances (mainly because of exchange broadening due to conformational exchange on the μs–ms time range), 18 were localized in the C-lobe, 5 on the linker region and neighboring residues, and only 8 in the N-lobe, suggesting that exchanges between different conformations in the complex take place on the μs–ms time range, particularly affecting the C-lobe, which establishes fewer contacts with CDZ, as well as the linker region. In holo-CaM, the backbone and CB chemical shifts of residues 77–81 of the interlobe linker are consistent with a highly dynamic segment, as indicated from chemical shift-derived S^2^ order parameters determined using Random Coil Index (RCI) algorithm [52] (Fig. S9). However, in the presence of CDZ, we observed a shift and exchange broadening of the corresponding amide signals (as well as those of neighboring residue signals 75–79 and 82–83), with residues 80D, 81S, 84E, and 74R displaying very strong CSPs (Fig. 4B).

Addition of an excess of CDZ to holo-CaM brings only minor modifications to the spectrum of holo-CaM bound to one CDZ, as evidenced by the very low CSP values observed between the holo-CaM:CDZ complexes at a molar ratio of 1:1 and 1:2.9 (Fig. 4B). This relatively small effect that might reflect partial or complete binding of a second CDZ molecule can also be rationalized considering that (i) the binding sites and residues in contact with CDZ are similar in both complexes for CDZ_A_ and CDZ_B_, (ii) several signals of residues close to CDZ_C_ were exchange broadened and could not be assigned (see Fig. 4 legend for details), and (iii) the structures of one- and two-CDZ loaded holo-CaM are similar.

Whereas binding of CDZ to holo-CaM is a slow process on the chemical shift time scale, ^1^H-^15^N spectra indicate that some residues undergo a faster chemical exchange within the complex, as revealed by very small chemical shift variations that depend on CDZ concentration observed for residues in the 2nd, 3rd, and 4th calcium binding loops, with residues such as 57A (2nd loop) and 137N (4th loop) showing the highest effect. Either fast conformational exchange within the complex or a modification of the in and out rate of calcium ions could be at the origin of the chemical shift variations.

### Influence of CDZ on the internal dynamics of holo-CaM

The internal dynamics of free and CDZ-bound holo-CaM were compared using the ^15^N transverse (T_2_), longitudinal (T_1_), and heteronuclear ^1^H-^15^N nuclear Overhauser effect (nOe) relaxation parameters (Figs. S10 and S11). In holo-CaM, the relaxation parameters (high T_2_, low T_1_, low T_1_/T_2_, and low ^1^H-^15^N nOes) of residues in the linker region (77–81) and flanking residues in α-helices H4 and H6 are characteristic of high amplitude motions on the ns-ps time scale, with a higher flexibility around its mid-point (T79-D80). In contrast, the relaxation parameters of the α-helical elements of the N- and C-lobes are indicative of ordered regions in a globular protein. Binding of CDZ drastically changes the internal dynamics of holo-CaM.

**Fig. 5:**
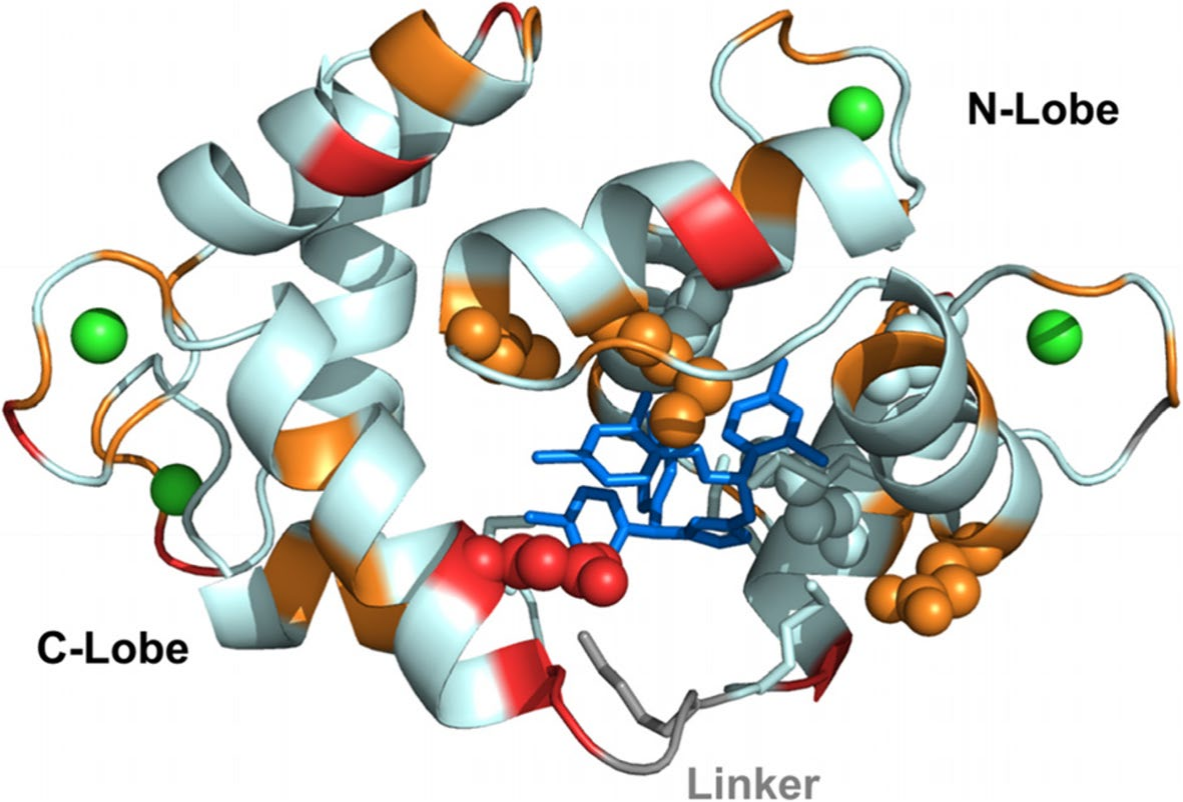
Decrease of backbone amide internal motion amplitudes in the nanosecond-picosecond time scale of holo-CaM upon binding to CDZ. The difference of the heteronuclear ^1^H-^15^N nOe of CDZ bound and free holo-CaM is color-coded on the cartoon representation of the holo-CaM:CDZ 1:1 X-ray structure. Red indicates very high positive nOe differences (≥ 0.18) and orange high nOe differences (between 0.10 and 0.16), denoting a decrease in the amplitude of fast internal motions (ns-ps) in the complex. CDZ is shown as blue sticks, Ca^2+^ ions are displayed as green spheres and the linker residues are shown in grey. The side chains of the CaM residues in close contact with CDZ as defined by Ligplot+ are highlighted as spheres if assigned (19, 36, 39, 54, 63, 71, 84) or as sticks (51, 72, 76, 77, and 145) if not observed by NMR

A simple picture of the modification of the amplitude of the fast motions caused by CDZ binding can be obtained from the difference of the nOe values between the bound and free conformations (Fig. 5). Most residues with available data displayed higher nOes within the complex, with 44 residues (highlighted in red and orange in Fig. 5) showing a significant CDZ-induced effect. These data suggest a global reduction of internal fast motions (ns-ps) of holo-CaM within the complex. Albeit residues in all the secondary structure elements and calcium binding loops showed this increase in nOe, the latter was not evenly distributed throughout the structure. Among calcium binding loops, loop 3 had the higher increase in nOe. This stabilizing effect was less pronounced for α-helix H1, which is far away from the CDZ binding site. Importantly, the increase of nOe for residues 80D and 81S in the linker was of 0.4, which indicates that CDZ binding restricts the fast motions in the linker. The fact that residues 77–79 in the linker and flanking residues 75–76 (unassigned) were highly impacted by CDZ binding and exchange-broadened further shows that the linker experiences conformational modifications and motion restrictions also on a slower time scale (μs–ms). This motion restriction is however modest, and the linker region remains dynamic in the complex as evidenced by HDX-MS that probes dynamics on a longer time scale (< minutes). As observed with CSPs, binding of a second CDZ molecule (sample with a three-fold excess of CDZ) resulted in modest modifications in the ^15^N T_1_, T_2_, and ^1^H-^15^N nOe profiles of holo-CaM relative to the 1:1 complex (Fig. S11). No clear evidence of different rigidity in the 1:1 or 1:2 complexes was detected. Taken together, the sub-nanosecond internal dynamics of holo-CaM:CDZ 1:1 and 1:2 complexes are similar (and reduced relative to the free protein), and holo-CaM experiences slow μs–ms conformational changes within the complex.

## Discussion

In the present work, we have explored the structural basis of holo-CaM interaction with CDZ, one of the most potent CaM antagonists known to date. CDZ has been widely used to probe the role of holo-CaM in various biological processes [22, 33, 34, 52]. How this molecule interacts with holo-CaM and inhibits its regulatory activity had not been elucidated at the molecular level. Although numerous studies have been carried out to define the biochemical and biophysical parameters of holo-CaM:CDZ interaction, major uncertainties remained regarding the structure, dynamics, affinity, and stoichiometry of the complex.

Our present results reveal a primary CDZ binding site involving mainly the N-lobe of CaM, although a second CDZ could be detected in crystals grown at high CDZ:holo-CaM ratio and high concentration (10 mM CDZ). Our NMR structural studies indicate that only a very low proportion of holo-CaM is complexed with two CDZ (holo-CaM:CDZ_BC_), which is unlikely to be of any relevance in physiological conditions, i.e*.*, when CDZ is used to probe the potential implication of CaM in signaling pathways and/or physiological processes (usually at concentrations below 20 μM). Earlier work, mainly based on indirect measurements using fluorescent probes, had speculated on multiple CDZ binding sites—up to six molecules per CaM [33], but our data clearly rule out such models.

Structures of other CaM:inhibitor complexes previously revealed that CaM may accommodate a variable number of antagonists in distinct calcium-loaded conformations, (e.g., 2 or 4 trifluoperazine (TFP) per CaM or 2 arylalkylamine antagonists, etc.), and even two distinct inhibitors simultaneously for example, trifluoperazine and KAR-2 or vinblastine and KAR-2, but not trifluoperazine and vinblastine [26]. This diversity may reflect the somehow artificial conditions of crystallization that use high concentrations of compounds (and CaM) and are likely favoring binding of these hydrophobic molecules to the hydrophobic cavities of CaM, a consideration that should be kept in mind when interpreting the corresponding crystal structures.

**Fig. 6:**
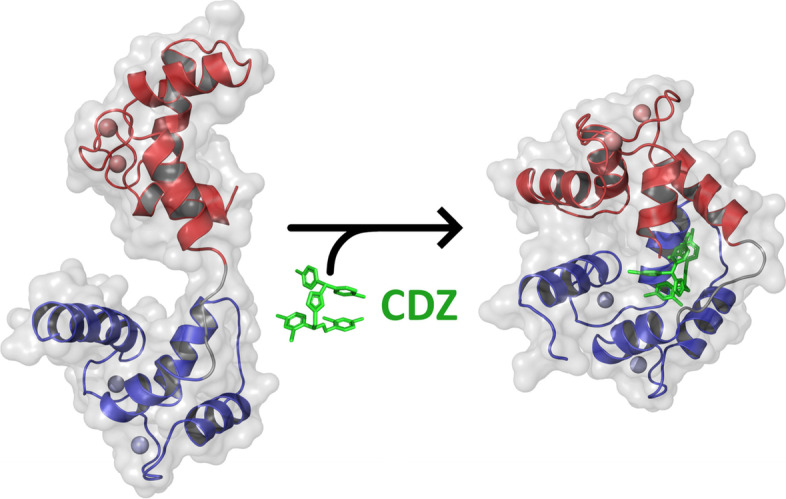
Effect of CDZ binding on calmodulin. CDZ binding dramatically affects the conformation of CaM, which collapses from a dumbbell-shaped conformation into a compact globular structure in which the CaM lobes wrap around CDZ. A single CDZ molecule is enough to induce dynamics and structural changes of CaM. The N-lobe and C-lobe of holo-CaM are depicted in blue and red, respectively

Our integrative structural biology approach indicates that CDZ binding dramatically affects the conformational dynamics of CaM that collapses from a dumbbell-shaped conformation into a compact globular structure in which the CaM lobes wrap around CDZ [7]. Despite the fact that CDZ mainly contacts the N-lobe, it induces a complete closing of the two lobes of holo-CaM into a structure that appears very similar to that of CaM bound to classical CBS peptides (e.g., MLCK [49, 53, 54]). The CDZ-induced compaction was clearly established both in crystal and in solution. The CDZ-induced stabilization of this closed conformation thus locks CaM into an inactive form unable to associate with most CBS of target proteins. Even a target enzyme, such as *B. pertussis* CyaA that is activated by interacting with the CaM C-terminal lobe only [49, 55, 56], can be efficiently inhibited by CDZ [35]. This suggests that, from a structural point of view, CDZ, by stabilizing the closed CaM globular conformation, should prevent CaM association with most enzyme targets (notwithstanding the relative CaM affinity of CDZ and CaM targets). Interestingly, this mode of binding is similar to that of TFP except that this antagonist, in contrast to CDZ, mainly contacts residues in the C-lobe of CaM [25]. Hence, in both cases, CDZ binding, primarily to one of the two lobes of holo-CaM, is sufficient to induce an open-to-closed conformational reorientation of the two domains of the protein (Fig. 6).

The two crystal structures of holo-CaM:CDZ with one and two CDZ molecules revealed that the key CDZ binding residues, mainly localized within the N-lobe of holo-CaM, are mostly hydrophobic and include several methionine residues. Interestingly, in the 1:2 complex, one of the two CDZ molecules (CDZ-B, with an occupancy 0.88) adopts essentially the same binding mode as the single CDZ (CDZ-A) in the 1:1 complex, although with a slightly different arrangement that is likely required for accommodating the second CDZ (with an occupancy of 0.82). NMR studies clearly indicated that, within the 1:1 holo-CaM-CDZ complex, some residues undergo exchange between different conformations on a slow (μs-ms) time scale. It is thus possible that in solution, CDZ is continuously sampling different configurations within the closed holo-CaM-CDZ globular complex, a feature that was also recently observed for another antagonist idoxifene [30]. Binding of a second CDZ molecule mainly onto the CaM C-lobe triggers very little changes on the overall structure of the complex and its internal dynamics. Structural analysis of holo-CaM-CDZ in solution is clearly in favor of a 1:1 stoichiometric complex. Overall, binding of a second CDZ is unlikely to occur in solution in standard experimental conditions in vitro or in cells (with CDZ below 20 μM) and it is therefore reasonable to assume that in these tests, binding of a single CDZ can fully block CaM activity. This is important to consider when experimentally probing CaM signaling function with CDZ at a concentration able to match the total CaM concentration in cell (estimated to range from 3 to 10 μM, depending on cell types, from which a large fraction of CaM may be bound to intracellular targets; see [57–59] and references therein).

Collectively, these data highlight the remarkable structural plasticity of CaM that can adopt highly diverse conformations to precisely fit to widely different antagonist scaffolds as well as to peptidic-binding motifs on target protein effectors. Our present results also open up new opportunities to design CDZ derivatives with substantially higher CaM-affinity, and consequently, better selectivity. Indeed, CDZ is known to affect several cellular targets, including L-type Ca^2+^, K^+^, Na^+^ channels, and sarcoplasmic reticulum (SR) calcium-release channels [60]. At high concentrations, it may exhibit other pharmacologic effects as well. CDZ is cytotoxic at high concentrations and has been shown to induce apoptosis in breast cancer and hepatoma cells [2, 61], to inhibit growth of murine embryonal carcinoma cells [62], and to enhance differentiation of colon cancer cells as well [2, 63]. These adverse effects somehow limit the application of this molecule (as well as that of all other CaM antagonists thus far) in dissecting calcium/CaM signaling. A more selective targeting of CDZ analogs onto holo-CaM could restrict the off-target effects and improve its biological relevance in fundamental research but also potentially in therapeutic applications [64–66]. Renewed efforts are currently applied to develop improved CaM-antagonists as illustrated by Okutachi et al. who recently described the development of a new covalent CaM inhibitor, called Calmirasone1, to explore the cancer cell biology of K-Ras and CaM associated stemness activities [67]. Such CaM inhibitors might find broad medical applications, as illustrated by Taylor and colleagues [68], who provided evidence that CaM inhibitors could be efficacious in rescuing a genetic disease (Diamond-Blackfan anemia, DBA) that results from increased expression of tumor suppressor protein 53. This study suggests that CaM inhibition may offer a potential therapeutic path for treatment of DBA and other diseases characterized by aberrant p53 activity [68, 69].

## Conclusions

In this work, we present an-in depth structural characterization of holo-CaM in complex with CDZ, a widely used inhibitor of this major calcium sensing protein. By combining SEC-SAXS, X-ray crystallography, HDX-MS, and NMR approaches, we delineate the molecular dynamics and structural changes of holo-CaM upon binding CDZ. We show that, although CDZ mainly contacts the N-terminal lobe of CaM, it stabilizes a compact globular structure in which the two lobes of the protein are wrapped around the inhibitor. This open-to-closed conformational reorientation of the two lobes of CaM is associated with a strong stabilization of its structural elements and reduction of protein dynamics, closely resembling the structural changes induced by natural CaM-binding peptides derived from protein targets. The dynamics and structural data presented herein will be useful to design the next generation of CDZ analogs with improved selectivity for CaM and restricted off-target effects.

## Methods

### Chemicals

Calmidazolium chloride (CDZ, CAS 57265-65-3, compound R_24571, M = 687.70 g/mol) was purchased from Merck (product reference 208665). CDZ was resuspended in 100% DMSO at a final concentration of 14.5 mM. CDZ was resuspended at 145 mM in 100% DMSO for the crystallography experiments of the holo-CaM:CDZ complex prepared at a molar ratio of 1:2. The MLCK, H-helix, and P454 peptides [35, 49, 70] were synthesized by Genosphere (Paris) and prepared as previously described [70].

### Buffers

Experiments were performed using the following buffers:

- Buffer A: 20 mM HEPES, 150 mM NaCl pH 7.4
- Buffer B: 20 mM HEPES, 150 mM NaCl, 2 mM CaCl_2_, pH 7.4
- Buffer C: 20 mM HEPES, 150 mM NaCl, 2 mM EDTA, pH 7.4
- Buffer D: 20 mM HEPES, 150 mM NaCl, 2 mM CaCl_2_, 2% DMSO, pD 7.4 prepared in 99.98 atom % D deuterium oxide
- Buffer E: 20 mM HEPES, 150 mM NaCl, 2 mM CaCl_2_, 2% DMSO and 20 μM CDZ, pD 7.4 prepared in 99.98 atom % D deuterium oxide
- Buffer F: 20 mM HEPES, 150 mM NaCl, 2 mM CaCl_2_, 5% DMSO, pH 7.4

### Calmodulin purification

Human Calmodulin (CaM) was produced in *E. coli* and purified as previously described [49, 56, 71]. Briefly, CaM was precipitated with ammonium sulfate followed by a glacial acetic acid precipitation. Then, CaM was purified as follows: a first HIC on Phenyl Sepharose (apo-CaM) in the presence of EDTA, an IEC on Q-Sepharose fast flow, a second HIC on Phenyl Sepharose (holo-CaM) in the presence of calcium (and eluted in EDTA), and a SEC on Sephacryl S100 equilibrated in buffer A. Calmodulin is stocked in buffer A. Protein concentration was determined by spectrophotometry (ε_280nm_ = 2,980 M^−1^ cm^−1^) and protein integrity was checked by SDS-PAGE and intact mass spectrometry analysis (M = 16706.03 gmol^−1^) by ESI-Q-ToF. The MS analysis indicates that the N-terminal methionine residue is post-translationally cleaved in *E. coli*.

### Isothermal titration calorimetry

ITC experiments were performed using a VP-ITC calorimeter (Malvern Panalytical, Orsay, France). The ITC experiments were performed in buffer F at 25°C (unless otherwise stated). For a typical titration, the solution of analyte (8 μM of holo-CaM) was loaded in the reaction cell. The titrant (200 μM of CDZ) was loaded into the syringe before injection of 5 to 15 μl of the titrant into the reaction cell at intervals of 600 s. Heats of dilutions were measured by injecting the titrant into the buffer and were subtracted from the heat of reaction. The titration profiles were analyzed using the Origin7 software (OriginLab, Northampton, MA, USA) to determine the thermodynamic parameters, as previously described [35].

### Synchrotron radiation circular dichroism

Synchrotron radiation circular dichroism (SRCD) was performed on the DISCO beamline of the synchrotron SOLEIL (Saint-Aubin, France). Spectra were recorded at 25°C with an integration time of 1.2 s and a bandwidth of 1 nm with a resolution of 1 nm. A far-UV spectrum represents the average of four individual scans. QS cells (Hellma, France) with a pathlength of 20, 50, 100, or 200 μm (depending on final protein concentration) were used to record spectra in the far-UV range (from 190 to 250 nm). CD spectra were measured in buffers A and B. The addition of DMSO, due to the use of CDZ resuspended in 100% DMSO, did not exceed 0.9% of DMSO in samples for SRCD. The CD unit is the mean residue ellipticity, MRE, expressed in (kilodegrees*cm^2^) / (dmol*residues), and calculated as previously described [56, 72].

### Small angle X-ray scattering

X-ray scattering data were collected at the SWING beamline [73] of the SOLEIL Synchrotron (Saint-Aubin, France) (Additional file 1: Table S1 gives all experimental details in accordance with BioSAXS publication guidelines [74]). Measurements were performed using a size-exclusion HPLC column (TSKgel G3000SW) online with the SAXS measuring cell, a 1.5-mm diameter quartz capillary contained in an evacuated vessel [75]. All samples were prepared in buffer F with 5–7% final DMSO (depending on CDZ concentration). Briefly, 50 μL of sample solution was loaded onto the equilibrated column. Scattering of the elution buffer before void volume was recorded and used as buffer scattering for subtraction from all protein patterns. Successive frames of 1 s were recorded. The elution flow of 0.5 mL/min ensured that no protein was irradiated for more than 0.4 s. Primary data reduction was performed using FOXTROT, the SWING in-house software. This yielded azimuthally averaged scattering intensities I(q) put on absolute scale (cm^−1^ units) using water scattering, where *q* is the momentum transfer (q = 4π sinθ/λ, where 2θ is the scattering angle and *λ* the wavelength of the X-rays). Data was subsequently processed using the program package PRIMUS [76] and RAW [77]. The forward scattering I(0) and the radius of gyration (Rg) were evaluated using the Guinier approximation [78]. Frames over the elution peak were analyzed individually before averaging the appropriate subset that yielded identical I(q)/c profiles with US-SOMO [79, 80]. The distance distribution function P(r) was determined using the indirect Fourier transform method as implemented in the program GNOM [81]. The program DENSS [37] was used to calculate electron density maps directly from scattering curves.

### Modelling holo-CaM using EOM

We undertook holo-Cam modeling in terms of ensembles of conformations using the package EOM (Ensemble Optimization Method) [38, 39]. EOM is a well-suited program suite to describe a flexible protein such as holo-CaM. The program Ranch within EOM creates a large (10,000) pool of random conformations, starting from the structures of the two calcium-bound lobes of holo-CaM (PDB ID: 1CLL). Residues (76–81) in the interlobe linker region were described as dummy residues. The program offers a choice between three chain types to be generated corresponding to the use of three Cα angle distributions: random coil, native, and compact. The native option was used. On average, random models will be more extended than native-like, while the “compact” option will force the reconstructed linkers to be rather compact. Dummy residues are subsequently substituted by complete residues using the programs PD2 [41] and SCWRL4 [42] before calculating all scattering patterns using Crysol [82]. The routine Gajoe within EOM tries to fit the experimental scattering curve by the average of the calculated scattering patterns of an ensemble of conformations using a genetic algorithm protocol. The program is run many times and yields equally good fits with different ensembles. The resulting conformations in any ensemble should therefore be considered as illustrations of the polypeptide chain main features, rather than actual conformations adopted by the protein.

### Crystallization of holo-CaM:CDZ complexes

The crystallization experiments were performed at 18°C by the sitting drop vapor diffusion technique in 96-well plates, according to established protocols at the Crystallography Core Facility of the Institut Pasteur [83]. For the initial screenings, sitting drops of 400 nL (1:1 protein to precipitant ratio) were set up in 96-well Greiner plates with a Mosquito automated nanoliter dispensing system (TTP Labtech, Melbourn, UK). The plates were then stored in a RockImager (Formulatrix, Bedford, USA) automated imaging system to monitor crystal growth. Initial crystallization hits were manually optimized by the hanging drop vapor diffusion technique in 24-well plates at 18 °C. The best crystals of the holo-CaM:CDZ complex were obtained by co-crystallization in hanging drops containing 1 mM of holo-CaM and 2.2 mM of CDZ in buffer B mixed with 2.8% DMSO, 30%w/v PEG 8K, and 0.2 M (NH_4_)_2_SO_4_ in the reservoir for the “1:1” complex and 1 mM of holo-CaM and 10.7 mM of CDZ in buffer B mixed with 7.4% DMSO, 0.2 M CaCl_2_, 0.1 M Tris pH 8.5 and 25 %w/v PEG 4K in the reservoir for the “1:2” complex. Crystals were then flash cooled in liquid nitrogen using the condition of crystallization supplemented with 30% (V/V) of ethylene glycol as cryoprotectant.

### Diffraction data collection and structure determination

Diffraction data were collected at beamlines PROXIMA 1 and PROXIMA 2A (synchrotron SOLEIL, St. Aubin, France) and processed with autoPROC [84]. The crystal structures of the complexes were solved by the molecular replacement method with Phaser [85], using separately the N-ter and C-ter lobes of holo-CaM as search models (both from the PDB entry 1CTR). Restrains for CDZ were generated using the Grade web server (http://grade.globalphasing.org/). Final models of the holo-CaM:CDZ complexes were obtained through interactive cycles of manual model building with Coot [86] and reciprocal space refinement with Buster [87]. Polder maps [88] were calculated using Phenix [89]. X-ray diffraction data collection and models refinement statistics are summarized in Additional file 1: Table S2. Figures were generated with PyMOL (version 2.5.2 The PyMOL Molecular Graphics System, Version 2.0 Schrödinger, LLC).

### Accession codes

Atomic coordinates and structure factors for the holo-CaM:CDZ complexes have been deposited in the RCSB Protein Data Bank under the accession code 7PSZ and 7PU9.

The molecular model and experimental SAXS data have been deposited on SASBDB (Small Angle Scattering Biological Data Bank, http://www.sasbdb.org/aboutSASBDB/) under the SASBDB ID SASDNX3 (calcium-bound calmodulin, including structural models) and SASDNY3 (calcium-bound calmodulin complexed with calmidazolium).

### HDX-MS experiments

#### Biological sample and chemical for HDX-MS

The initial CaM stock solution was at 276 μM in buffer A. The holo-CaM working solution was prepared at 10 μM in buffer B and incubated for 30 min at room temperature before use. Initial stock solution of CDZ at 14.5 mM in 100% DMSO. Working solution prepared at 1 mM in 100% DMSO and stored at -20°C.

#### Sample preparation for HDX-MS

A summary of the HDX data is provided in Additional file 1: Table S4, following recommendations for HDX-MS reporting [90]. The labelling was performed at room temperature using two distinct deuterated buffers (D and E) prepared in 99.98% deuterium oxide.

The holo-CaM:CDZ complex was formed by mixing 9.4 μL of holo-CaM (10 μM) with 0.6 μL of CDZ at 1 mM in a final volume of 30 μL buffer B. A CDZ-free holo-CaM control was prepared in parallel by replacing CDZ by DMSO. After 1-h incubation at room temperature, continuous labeling was initiated by adding 120 μL of deuterated buffer to 30 μL of each equilibrated protein solution (i.e., CDZ-free holo-CaM with Buffer D, CDZ-bound holo-CaM with Buffer E). The final deuterium excess was ~80% to favor unidirectional exchange. The DMSO concentration was maintained at 2% during labeling. The exchange reaction was quenched after 10 s, 20 s, 30 s, 1 min, 10 min, 30 min, and 120 min by mixing 20 μL of the labeling reaction (i.e., 12.54 pmol of holo-CaM) with 40 μL of quench buffer (2.5% formic acid, 4M urea) maintained at 4°C to achieve a final pH of 2.5 (final D_2_0/H_2_0 ratio: 0.27/0.73). Quenched samples were immediately snap-frozen in liquid nitrogen and stored at −80 °C. Undeuterated controls were treated using an identical procedure. Triplicate labeling experiments were performed for each time point and condition for all HDX-MS analyses (independent technical replicates).

#### HDX-MS data acquisition

HDX-MS analyses were performed with the aid of an HDX manager (Waters Corporation, Miliford, MA) maintained at 0 °C. Prior to mass analysis, samples were rapidly thawed and 10.45 pmol (i.e., 50 μL) of labeled holo- or apo-CaM (with or without CDZ) were digested for 2 min at 20 °C using an in-house packed immobilized pepsin column (2.0 × 20 mm, 66 μL bead volume; immobilized pepsin from Thermo Scientific). Peptides were trapped, concentrated, and desalted using a VanGuard™ CSH C18 pre-column (1.7 μm, 2.1 × 5 mm; Waters Corporation, Miliford, MA), and separated using an ACQUITY UPLC™ CSH C18 column (1.7 μm, 1 × 100 mm, Waters Corporation, Miliford, MA). Labeled peptides were separated over an 8-min gradient of 5–30% acetonitrile at 40 μL/min and 0°C. After each run, the pepsin column was manually cleaned with two consecutive washes of 1.5% formic acid, 5% acetonitrile, 1.5 M guanidinium chloride, pH 1.6. Blank injections were performed after each sample to confirm the absence of peptide carry-over.

The LC flow was directed to a Synapt™ G2-Si HDMS™ mass spectrometer (Waters Corporation) equipped with a standard electrospray ionization source (ESI). Mass accuracy was ensured by continuously infusing a Glu-1-Fibrinogen solution (100 fmol/μL in 50% acetonitrile, 0.1% formic acid) through the reference probe of the ESI source at a flow rate of 3 μL/min. Mass spectra were acquired in positive-ion and resolution mode over the 50–1950 m/z range. CaM peptic peptides were identified in undeuterated samples by a combination of data independent acquisition (MS^E^) and exact mass measurement (below 10 ppm mass error) using the same chromatographic conditions than for the deuterated samples. Four distinct MS^E^ trap collision energy ramps were employed to optimize the efficiency of the fragmentation: 10–30V (low), 15–35V (medium), 20–45V (high), and 10–45V (mixed mode).

#### HDX-MS data processing

DynamX 3.0 (Waters Corporation, Miliford, MA) was used to extract the centroid masses of all peptides selected for local HDX-analyses; only one charge state was considered per peptide. The final peptide map of CaM was refined in DynamX 3.0 using the following Protein Lynx Global Server (PLGS) import options: minimum intensity: 3000; sum of products per amino acid: 0.15; sum of intensity for products: 1000; minimum PLGS score: 6.5; maximum MH+ error (ppm): 5; file threshold: 2. A total of 40 peptides covering 93.2% of the CaM sequence were selected for HDX-MS based on their signal intensity and signal over noise ratio (Additional file 1: Fig. S3). No adjustment was made for back-exchange; results are therefore reported as relative deuterium exchange levels expressed in either mass unit or fractional exchange. Overlapping CaM peptides were only used to improve the spatial resolution if their back exchange values were identical and ≤ at 10% (Note: the fully deuterated CaM sample was acquired in a previous study using identical experimental conditions). Fractional exchange data was calculated by dividing the experimentally measured uptake by the theoretically maximum number of exchangeable backbone amide hydrogens that could be replaced into each peptide (taking into account the final excess of deuterium present in the labeling mixture). MEMHDX was used to visualize and statistically validate HDX results (Wald test, false discovery rate sets to 5%) [91].

### Nuclear magnetic resonance

Samples of ^15^N or ^15^N/^13^C labeled holo-CaM (Giotto Biotech, Italy) were prepared in 20 mM HEPES pH 7.0, 100 mM NaCl, 2 mM CaCl_2_, supplemented with appropriate amounts of D_2_O/DMSO-d6 (Eurisotop, France). Protein concentration ranged between 30 and 270 μM. Due to the poor solubility of CDZ, all NMR experiments were performed with a final free-CDZ concentrations below 200 μM. For holo-CaM-CDZ interaction studies, CDZ dissolved in DMSO-d6 was added to holo-CaM samples. The final DMSO-d6 concentration varied between experiments but was always lower than 7%. We checked by using NMR ^1^H-^15^N correlation spectroscopy that holo-CaM spectra, and hence the protein structure and dynamics, were not affected by DMSO up to 10% concentration.

NMR experiments were run on a 600 MHz Avance III HD spectrometer (Bruker BioSpin, Billerica, USA) equipped with a triple resonance (^1^H/^13^C/^15^N) cryogenically cooled probe. Spectra were recorded with Topspin 3.6.3 (Bruker), processed with NMRPipe [92], and analyzed with CCPNMR analysis 2.4.3 [93]. Experiments were performed at 37°C and referenced to the sodium salt of 4,4-dimethyl-4-silapentane-1-sulfonic acid.

Backbone (^1^H,^15^N, CA) and CB chemical shifts of the free (260 μM) and CDZ bound forms (265 μM) of holo-CaM were assigned using standard two- and three-dimensional experiments: ^13^C and ^15^N HSQC (heteronuclear single quantum coherence) [94], ^1^H-^15^N SOFAST-HMQC, and the BEST (band-selective excitation short transient) versions of the HNCACB, CBCA(CO)NH, HNCA, and HN(CO)CA pulse sequences implemented in NMRLib 2.0 [95]. While for free holo-CaM, all residues could be assigned, for the holo-CaM:CDZ complex, there were 11 missing resonances in ^1^H-^15^N correlation spectra and 22 were shifted and typically too broad to permit assignment.

Secondary structures of the free and bound forms were estimated from backbone ^1^H,^15^N, CA and side chain CB chemical shifts using Talos-N [96]. The secondary structures of holo-CaM thus obtained were compared to the secondary structures of holo-CaM ([38], PDB ID 1J7O and 1J7P for the N- and C-lobes, respectively) and holo-CaM:CDZ complexes determined in this work as established by DSSP [97].

Backbone and CB chemical shifts were also used to calculate the order parameter (S^2^) of isolated holo-CaM to analyze the amplitude of motions on the ns-ps time range. S^2^ values were determined using the RCI software [52].

The chemical shift perturbation (CSP) upon CDZ binding to holo-CaM was determined from the differences of ^15^N (∆δ_N_) and ^1^HN (∆δH_N_) chemical shifts of the free and bound forms using the formula:

$$CSP=\sqrt{{\left(0.159\times \Delta {\updelta}_N\right)}^2+\Delta {\updelta}_{HN}^2}$$

The translation (d) and rotational diffusion (τ_c_) of holo-CaM and the effect of CDZ binding at different holo-CaM:CDZ ratios were evaluated on ^15^N labeled holo-CaM samples (± CDZ) by ^1^H detected 1D ^15^N-edited ^1^H pulsed-field gradient self-diffusion experiments coupled to the ^1^H-^15^N SOFAST-HMQC sequence, and by ^15^N cross-correlated spin relaxation experiments (TRACT, TROSY for rotational correlation times [98]) as implemented in NMRLib 2.0 [95] (N15_TRACT_1D sequence). To consider the effect on viscosity of different D_2_O and DMSO concentrations, results were extrapolated to 100% H_2_O using the viscosities of the corresponding H_2_O, D_2_O, and DMSO mixtures. Briefly, to determine the τ_c_ value, we evaluated the fast (R_β_) and slow (R_α_) ^15^N cross-relaxation rates by fitting to exponential decays the integral of the whole amide envelope from ^1^H - 1D spectra obtained using 10 different relaxation times and a 0.4 s recycling delay. Individual point errors were obtained by multiplying the noise standard deviation by the number of integrated points. Rate errors and confidence levels were obtained using 1000 Monte Carlo simulations. The τ_c_ value was determined by numerical (Powell) minimization of the theoretical and experimental (R_β_ − R _α_) difference as described in [98]. Errors in τ_c_ were also estimated through 1000 Monte Carlo simulations. Fits, minimizations, and Monte Carlo simulations were performed with in-house Python scripts.

The internal dynamics of holo-CaM were analyzed by determining the ^15^N longitudinal (T_1_) and transverse (T_2_) relaxation rates and the steady state ^1^H-^15^N heteronuclear nOe (nuclear Overhauser enhancement) relaxation parameters from 2D ^1^H-^15^N experiments [99]. The concentration of holo-CaM was 260 μM (5% D_2_O) for the free protein, 265 μM for the 1:1, and 65 μM for the 1:3 holo-CaM:CDZ samples (4% DMSO). Series of 5-6 spectra with a 5-s recovery delay between scans and different relaxation delays were recorded for T_1_ and T_2_ measurements. The ^1^H-^15^N nOe experiments were run with a recovery delay of 3 s and a saturation/no saturation delay of 4 s. Measurement errors were estimated from the spectra noise standard deviation. Fits to exponential decays and Monte Carlo evaluation of fit errors and confidence levels were performed with in-house Python scripts.

### Statistical analysis of the experimental datasets

2D-SAXS data were radially averaged, normalized to the intensity of the incident beam, and put on an absolute scale using the scattering from water [100] before buffer scattering subtraction. All these operations were performed using the programs FoxTrot (courtesy of SWING beamline) and Primus (https://www.embl-hamburg.de/biosaxs/primus.html) [101]. The resulting 1D scalar scattering intensity profiles are represented by the normalized intensities and their associated standard deviations (SD). Identical frames under the main elution peak were selected using Cormap [102] and averaged for further analysis. The agreement between experimental data and calculated intensities from models was evaluated using the reduced *χ*^2^ metric [43].

X-ray data collection, processing, and model refinement statistics corresponding to the X-ray structures are summarized in Additional file 1: Table S2. The software packages used are autoPROC, Phaser, Coot, Buster, and Pymol, as described in the Crystallography section.

The statistical analysis for the HDX-MS experiments is described below. A summary of the HDX-MS experiments is provided in Additional file 1: Table S4 and in the “HDX-MS analysis of holo-CaM upon CDZ binding” section. Pre-processing of data: one unique charge state was considered per peptide (user selection). The quality determination of the dataset was accomplished by MEMHDX (http://memhdx.c3bi.pasteur.fr) to evaluate the agreement across replicates. Data presentation: Logit representation is used for Additional file 1: Fig. S12.

For Additional file 1: Fig. S12, each dot reported on the uptake differential plots corresponds to the average value of three independent replicates. The repeatability of the measurement determined for each state (pooled standard deviation) using the 46 selected peptides and the 966 unique MS data points per state is reported in Additional File 1: Table S4. Sample size (*n*) for each statistical analysis: Triplicate labeling experiments were performed for each time point (7 time points including unlabeled controls) and condition for all HDX-MS analyses (independent technical replicates). Considering 46 peptides, 1 charge state, 3 replicates, 7 time points, and 2 conditions, the complete HDX-MS datasets contains *n*= 1932 unique data points. Statistical methods used to assess significant differences with sufficient details: To consider the time dependency of the exchange reaction, two distinct *p*-values were calculated per peptide using two individual Wald tests. The FDR was set to 5% (*p* < 0.05). Software used for statistical analysis: MEMHDX (http://memhdx.c3bi.pasteur.fr).

The statistical analysis for NMR experiments was performed as follows. Measurement errors were estimated from the spectra noise standard deviation. Fits to exponential decays and Monte Carlo evaluation of fit errors and confidence levels were performed with in-house Python scripts. Error bars were obtained from 1000 Monte Carlo simulations considering one noise standard deviation.

## Supplementary Information

**Additional file 1: Figure S1.** Synchrotron radiation far-UV circular dichroism. **Figure S2.** Isothermal titration calorimetry of holo-CaM:CDZ. **Figure S3.** Analysis of holo-CaM using SAXS. **Table S1.** SAXS data collection and scattering derived parameters. **Table S2.** Crystallographic data collection and refinement statistics. **Figure S4.** Difference electron density map. **Figure S5.** Interactions between CDZ and holo-CaM residues visualized using Ligplot+. **Table S3.** Detailed location of residues interacting with CDZ according to Ligplot+ and comparison with HDX-MS data. **Table S4.** HDX-MS data summary. **Figure S6.** Peptide map of CaM by HDX-MS. **Figure S7.** Deuterium uptake plots. **Figure S8.** Comparison of the differential HDX patterns within holo-CaM upon H-helix (A), P454 (B), MLCK (C) and CDZ (D) binding. **Table S5.** Rotational correlation time (τ_c_) of holo-CaM at different holo-CaM:CDZ ratios. **Table S6.** Comparison of secondary structure content from SRCD, NMR and X-ray. **Figure S9.** Order parameter (S^2^) reflecting the amplitude of motions on the ns-ps time scales of free holo-CaM. **Figure S10.** holo-CaM internal dynamics from ^15^N relaxation data. **Figure S11.** Comparison of holo-CaM internal dynamics from ^15^N relaxation data in complex with CDZ. **Figure S12.** Statistical analysis performed with MEMHDX.

**Additional file 2.** The HDX-MS data

## Data Availability

All data generated or analyzed during this study are included in this published article, its supplementary information files, and publicly available repositories. The crystal structures have been deposited on the PDB with the access codes 6YNU and 6YNS. The structural models and experimental SAXS data have been deposited on SASBDB (Small Angle Scattering Biological Data Bank, http://www.sasbdb.org/aboutSASBDB/) under the SAS codes SASDNX3 (Calcium-bound Calmodulin, including structural models) and SASDNY3 (Calcium-bound Calmodulin complexed with Calmidazolium). All relevant HDX-MS, X-ray, and SAXS data are available in the “Methods” section and in the Additional file. The NMR data have been deposited on the BMRB with the access codes 51526 (Backbone chemical shifts of calcium-loaded human calmodulin at pH 7 and 37degC), 51527 (Calcium-loaded human calmodulin in complex with the antagonist calmidazolium), 51529 (Relaxation parameters of calcium loaded human calmodulin at pH 7.0 and 37degC (600 MHz)), and 51530 (Relaxation parameters of calcium loaded human calmodulin in complex with the antagonist calmidazolium at pH 7.0 and 37degC (600 MHz)). The HDX-MS data are available as Additional file 2: HDX-MS data. HDX-MS and NMR raw data are available upon request. The SRCD data have been deposited on the PCDDB.

## Abbreviations

BMRB: Biological Magnetic Resonance Data Bank
CaM: Calmodulin
C-lobe: C-terminal domain of CaM
CDZ: Calmidazolium
HDX-MS: Hydrogen/deuterium exchange mass spectrometry
holo-CaM: Calcium-loaded calmodulin
MEMHDX: Mixed-effects model for HDX experiments
MLCK: Myosin light chain kinase
MS: Mass spectrometry
N-lobe: N-terminal domain of CaM
NMR: Nuclear magnetic resonance
pdb: Protein Data Bank
SASBDB: Small Angle Scattering Biological Data Bank
SAXS: Small-angle X-ray scattering
SEC: Size-exclusion chromatography
