# Supplementary material for: Dynamics and structural changes of calmodulin upon interaction with the antagonist calmidazolium
Source: BMC Biol. 2022 Aug 9;20:176. doi: 10.1186/s12915-022-01381-5 (PMC9361521; doi:10.1186/s12915-022-01381-5)
Supplement: Supplementary file 1 — Additional file 1: Figure S1. Synchrotron radiation far-UV circular dichroism. Figure S2. Isothermal titration calorimetry of holo-CaM:CDZ. Figure S3. Analysis of holo-CaM using SAXS. Table S1. SAXS data collection and scattering derived parameters. Table S2. Crystallographic data collection and refinement statistics. Figure S4. Difference electron density map. Figure S5. Interactions between CDZ and holo-CaM residues visualized using Ligplot+. Table S3. Detailed location of residues interacting with CDZ according to Ligplot+ and comparison with HDX-MS data. Table S4. HDX-MS data summary. Figure S6. Peptide map of CaM by HDX-MS. Figure S7. Deuterium uptake plots. Figure S8. Comparison of the differential HDX patterns within holo-CaM upon H-helix (A), P454 (B), MLCK (C) and CDZ (D) binding. Table S5. Rotational correlation time (τc) of holo-CaM at different holo-CaM:CDZ ratios. Table S6. Comparison of secondary structure content from SRCD, NMR and X-ray. Figure S9. Order parameter (S2) reflecting the amplitude of motions on the ns-ps time scales of free holo-CaM. Figure S10. holo-CaM internal dynamics from 15N relaxation data. Figure S11. Comparison of holo-CaM internal dynamics from 15N relaxation data in complex with CDZ. Figure S12. Statistical analysis performed with MEMHDX. [file 12915_2022_1381_MOESM1_ESM.pdf]

Dynamics and structural changes of calmodulin upon interaction with the antagonist calmidazolium

Combined Additional Files 1: Figures S1-S12 – Table S1-S6

FigS1 - Synchrotron radiation far-UV circular dichroism. **FigS2 -** Isothermal titration calorimetry of Holo-CaM:CDZ. **FigS3 -** Analysis of Holo-CaM using SAXS. **TableS1 -** SAXS data collection and scattering derived parameters. **TableS2 -** Crystallographic data collection and refinement statistics. **FigS4 -** Difference electron density map. **FigS5 -** Interactions between CDZ and Holo-CaM residues visualized using Ligplot+. **TableS3 -** Detailed location of residues interacting with CDZ according to Ligplot+ and comparison with HDX-MS data. **TableS4 -** HDX-MS data summary. **FigS6 -** Peptide map of CaM by HDX-MS. **FigS7 -** Deuterium uptake plots. **FigS8 -** Comparison of the differential HDX patterns within Holo-CaM upon H-helix (A), P454 (B), MLCK (C) and CDZ (D) binding. **TableS5 -** Rotational correlation time (τ_c) of Holo-CaM at different Holo-CaM:CDZ ratios. **TableS6 -** Comparison of secondary structure content from SRCD, NMR and X-ray. **FigS9 -** Order parameter (S^2) reflecting the amplitude of motions on the ns-ps time scales of free Holo-CaM. **FigS10 -** Holo-CaM internal dynamics from ^{15}N relaxation data. **FigS11 -** Comparison of Holo-CaM internal dynamics from ^{15}N relaxation data in complex with CDZ. **FigS12 -** Statistical analysis performed with MEMHDX.

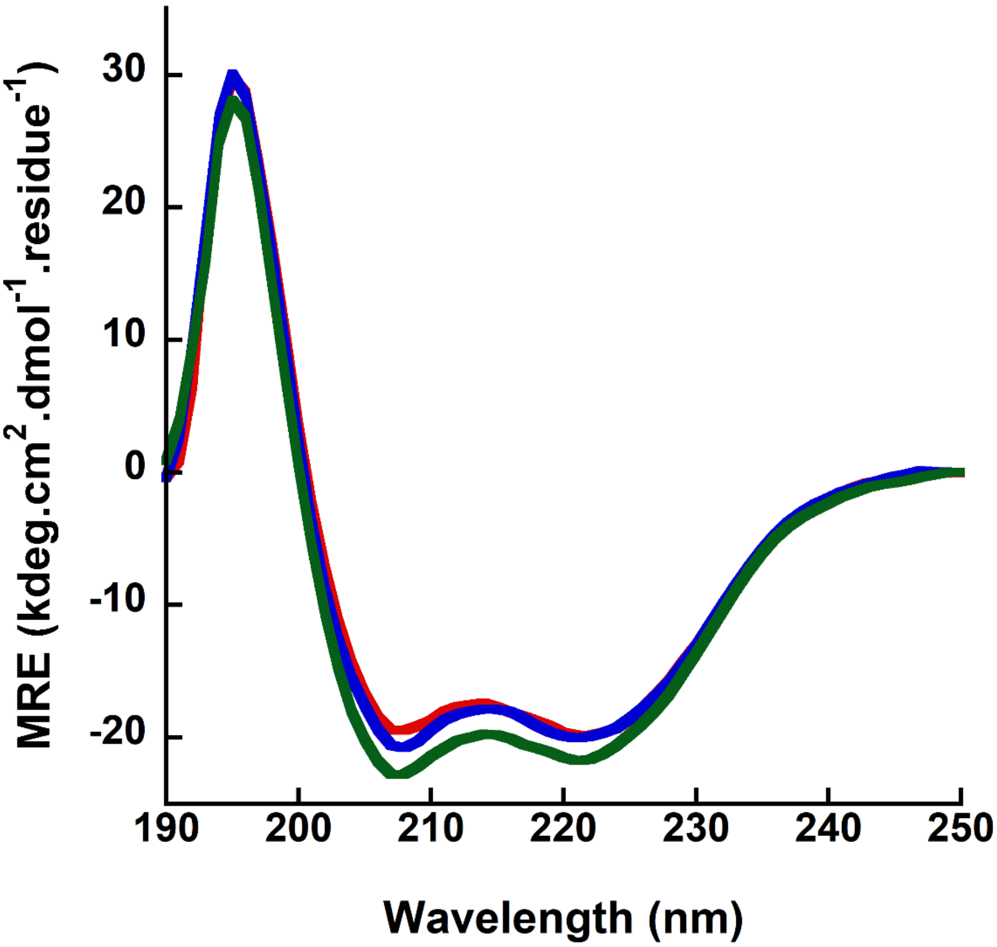

Figure S1: **Synchrotron radiation far-UV circular dichroism.** Holo-CaM (60 µM) in buffer B with 0.91% final DMSO in the absence (red) and in the presence of 66 µM of CDZ (blue, Holo-CaM:CDZ molar ratio of 1:1.1) or 132 µM of CDZ (green, Holo-CaM:CDZ molar ratio of 1:2.2).

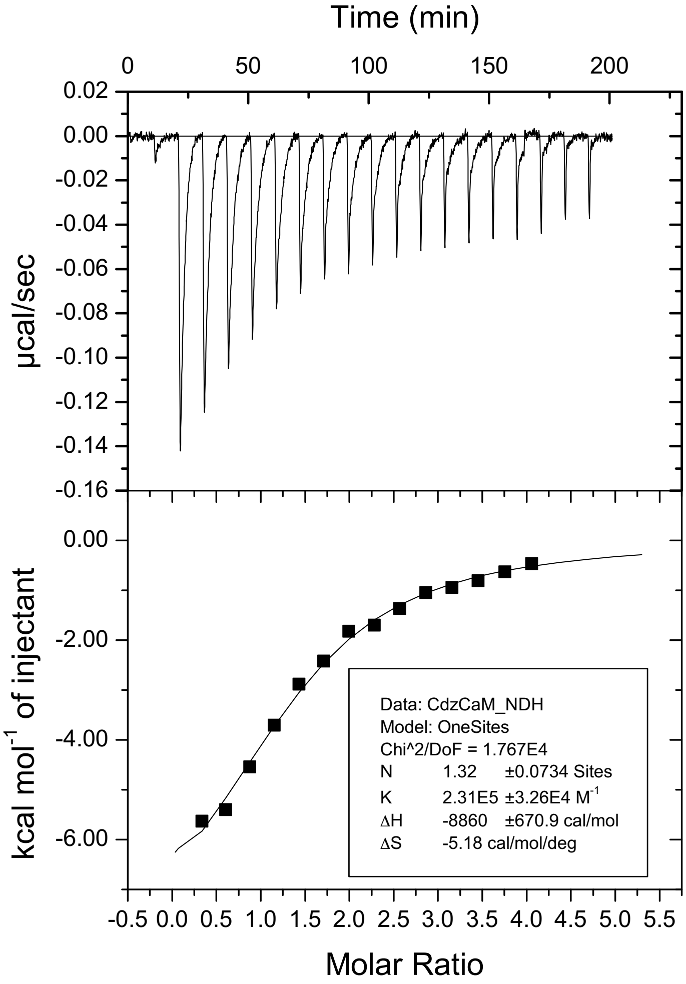

Figure S2: **Isothermal titration calorimetry of Holo-CaM:CDZ.** A solution of 8 µM Holo-CaM was loaded in the reaction cell. A solution of 200 µM CDZ was loaded into the syringe and then injected into the reaction cell. Heats of dilutions were measured by injecting 200 µM of CDZ into buffer B and were subtracted from the heat of reaction of the Holo-CaM titration by CDZ. The titration profiles were analyzed using the Origin7.0 software (OriginLab) to determine the thermodynamic parameters. In the example shown in this figure, the dissociation constant, K_D, is 4.3 µM and the stoichiometry is 1.3. The average values of K_D and stoichiometry (n=6 experiments) are 3 ± 2 µM and 1.2 ± 0.5, respectively.

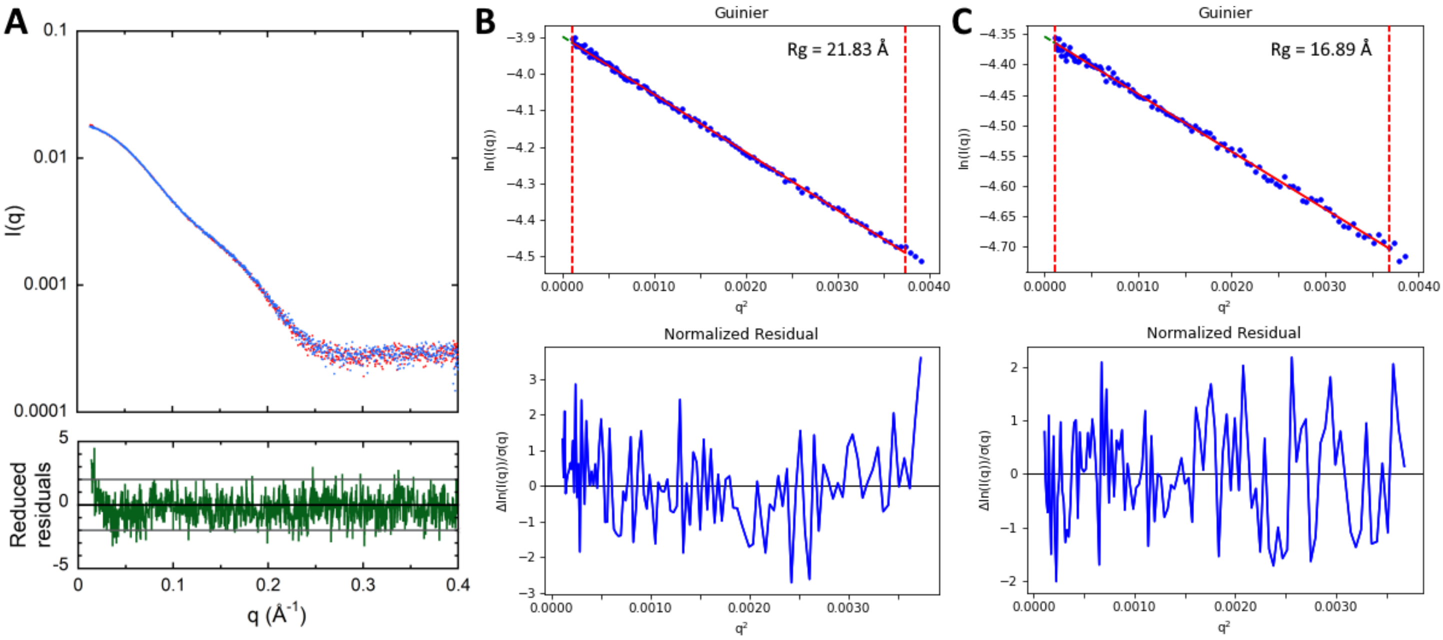

Figure S3: Analysis of Holo-CaM using SAXS. A. Scattering patterns of Holo-CaM in buffer B in the absence (red) and in the presence of 5% DMSO (blue) together with the reduced residuals distribution (green line). **B. and C.** Guinier fit and residuals for Holo-CaM in buffer B in the absence (left) or in the presence (right) of three molar excess of CDZ.

**Data collection parameters**			
Instrument	Beamline SWING (Synchrotron SOLEIL)		
Detector	EigerX4M in vacuum (Dectris)		
Sample to detector distance	2.00 m		
Beam geometry	400 µm x 200 µm		
Wavelength (Å)	1.033		
q-range ($Å^{-1}$)	$0.0041 < q < 0.50$		
Exposure time (ms) / reading time (ms)	990 / 10		
Temperature (K)	288		
Sample	Holo-CaM	Holo-CaM (prepared in 5% DMSO)	Holo-CaM:CDZ (prepared in 7% DMSO)
Molar extinction coefficient ($M^{-1}.cm^{-1}$)	2 980		
CaM molecular mass[(1)] (Da)	16 706		
Initial CaM concentration (µM)	500	500	333
Initial CDZ concentration (µM)	0	0	1050
Running buffer of the SEC	Buffer F		
**Structural parameters**			
I(0) Guinier (cm^{-1})	0.0182	0.0203	0.0129
R_g Guinier (Å)	21.98 ± 0.06	21.83 ± 0.03	16.89 ± 0.04
I(0) P(r) (cm^{-1})	0.183	0.0204	0.0129
R_g P(r) (Å)	22.49 ± 0.03	22.39 ± 0.04	16.83 ± 0.03
D_{max} (Å)	74	72	52
**Data reduction and analysis software**			
FOXTROT, ATSAS, RAW, US-SOMO, GNOM			
**Modeling software**			
DENSS, EOM			

Table S1: SAXS data collection and scattering derived parameters.

(1) Molecular mass of calmodulin in the absence of the N-terminal methionine residue. The expected averaged molecular mass from Protpi server (https://www.protpi.ch/Calculator/ProteinTool) is 16 706.25 Da. The averaged molecular mass measured by mass spectrometry is 16 706.18 +/-0.07 Da (delta = 0.07 Da, *i.e.*, 4.2 ppm).

Complexes	Holo-CaM:CDZ (molar ratio of 1:2.2)	Holo-CaM:CDZ (molar ratio of 1:10)
**Beamline**	PROXIMA-2A	PROXIMA-1
**Wavelength**	0.9801	0.8266
**Resolution range**	30.91 - 1.898 (1.966 - 1.898)	34.08 - 2.279 (2.36 - 2.279)
**Space group**	C 1 2 1	P 6_1 2 2
**PDB ID**	7PSZ	7PU9
**Unit cell**	66.848 36.127 68.382 90 116.738 90	39.351 39.351 336.92 90 90 120
**Total reflections**	78268 (7850)	278336 (29794)
**Unique reflections**	11450 (1097)	8006 (751)
**Multiplicity**	6.8 (7.2)	34.8 (39.7)
**Completeness (%)**	97.55 (96.39)	99.93 (99.87)
**Mean I/sigma(I)**	17.21 (2.57)	17.84 (2.71)
**Wilson B-factor**	37.33	52.14
**R-merge**	0.06148 (0.8184)	0.1477 (1.307)
**R-meas**	0.06686 (0.8823)	0.15 (1.324)
**R-pim**	0.02585 (0.3274)	0.02589 (0.2087)
**CC1/2**	0.998 (0.906)	0.999 (0.909)
**CC***	1 (0.975)	1 (0.976)
**Reflections used in refinement**	11450 (1095)	8006 (750)
**Reflections used for R-free**	583 (45)	406 (25)
**R-work**	0.2281 (0.3077)	0.2341 (0.2600)
**R-free**	0.2574 (0.3430)	0.2663 (0.2775)
**CC(work)**	0.947 (0.802)	0.904 (0.862)
**CC(free)**	0.946 (0.442)	0.932 (0.902)
**Number of non-hydrogen atoms**	1281	1259
**macromolecules**	1149	1134
**ligands**	89	84
**solvent**	43	41
**Protein residues**	145	144
**RMS(bonds)**	0.011	0.014
**RMS(angles)**	1.45	1.48
**Ramachandran favored (%)**	99.30	97.89
**Ramachandran allowed (%)**	0.70	2.11
**Ramachandran outliers (%)**	0.00	0.00
**Rotamer outliers (%)**	1.59	6.50
**Clashscore**	7.35	3.51
**Average B-factor**	45.22	60.49
**macromolecules**	43.24	60.53
**ligands**	69.12	59.36
**solvent**	48.66	61.64

Table S2: Crystallographic data collection and refinement statistics.
Statistics for the highest-resolution shell are shown in parentheses.

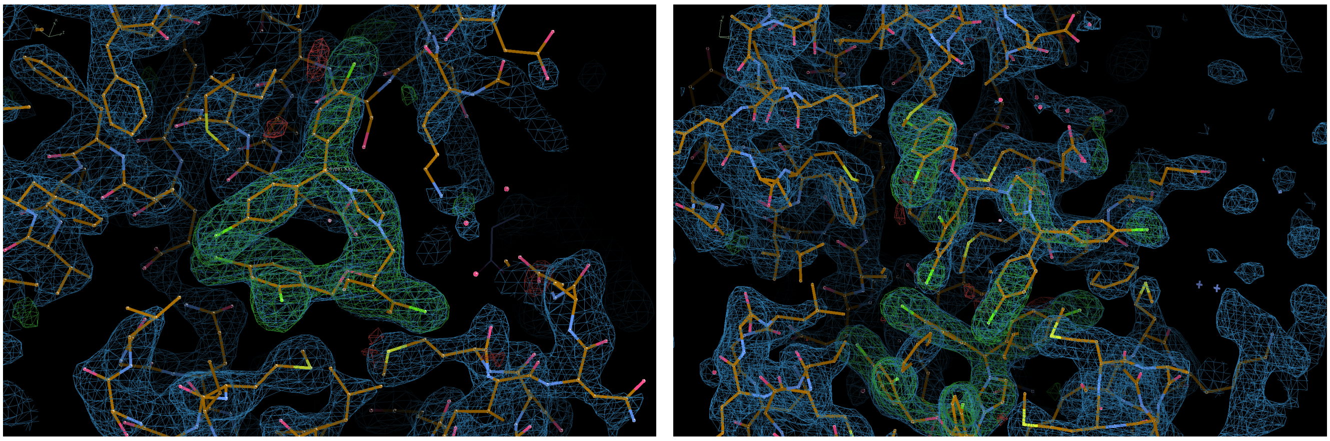

Figure S4: Difference electron density map. $2mF_o–DF_c$ and $mF_o–Df_c$ difference electron density maps (colored in blue and green, respectively) for PDB structure 7PSZ, calculated by setting the occupancy of the CDZ molecules to zero. In both panels maps are contoured at 1.2 and 3σ levels, respectively.

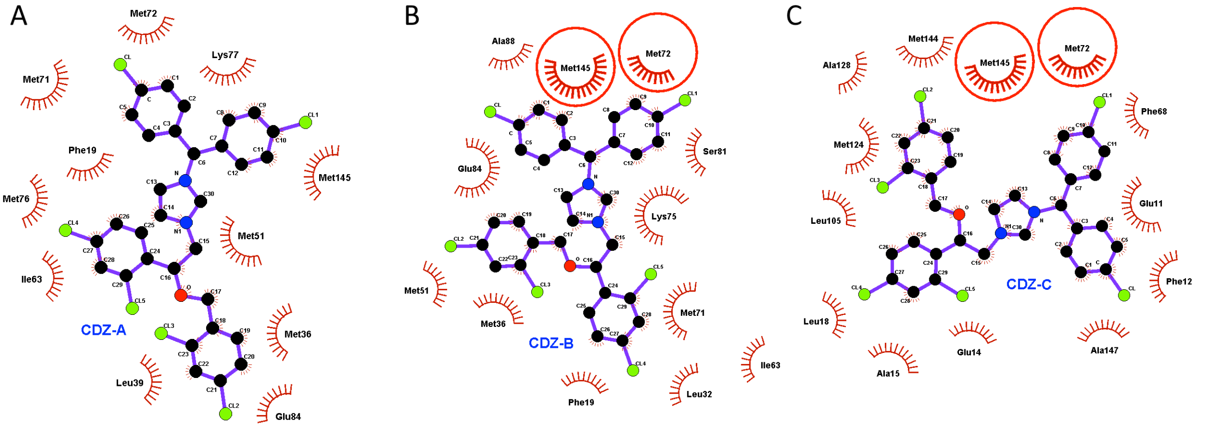

Figure S5: **Interactions between CDZ and Holo-CaM residues visualized using Ligplot+**. **A**. Ligplot of CDZ from the crystallographic structure of the 1:1 Holo-CaM:CDZ complex (PDB ID: 7PSZ) and its interacting residues. Most of these residues are located in the C-terminal part of N-CaM and in the vicinity of the central linker (residues 77-81). **B. and C.** Ligplot of the two CDZ molecules from the Holo-CaM:CDZ 1:2 crystallographic structure (PDB ID: 7PU9). Whereas CDZ-B interacts with almost the same residues than CDZ-A mainly located in the N-terminal lobe, CDZ-C interacts with residues in both lobes. Methionine residues 72 and 145 (circled in red) interact with both CDZ molecules, as well as with CDZ-A in the 1:1 complex. The vast majority of the Holo-CaM:CDZ interactions are hydrophobic.

	Holo-CaM:CDZ 1:1	Holo-CaM:CDZ 1:2		Effect in HDX-MS
	CDZ A	CDZ B	CDZ C	
N-Lobe			11 Glu	
			12 Phe	
			14 Glu	
			15 Ala	
			18 Leu	
	19 Phe	19 Phe		
		32 Leu		
	36 Met	36 Met		
	39 Leu			
	51 Met	51 Met		
	63 Ile	63 Ile		
			68 Phe	
	71 Met	71 Met		
	72 Met	72 Met	72 Met	
Linker region		75 Lys		
	76 Met			
	77 Lys			
		81 Ser		
C-Lobe	84 Glu	84 Glu		
		88 Ala		
			105 Leu	
			124 Met	
			128 Ala	
			144 Met	
	145 Met	145 Met	145 Met	
			147 Ala	

Table S3: Detailed location of residues interacting with CDZ according to Ligplot+ and comparison with HDX-MS data. CDZ-A is the CDZ molecule from the crystallographic structure of the 1:1 Holo-CaM:CDZ complex (PDB ID: 7PSZ, Holo-CaM:CDZ$_A$). CDZ-B and CDZ-C are the CDZ molecules from the crystallographic structure of the 1:2 Holo-CaM:CDZ complex (PDB ID: 7PU9, Holo-CaM:CDZ$_{BC}$). The effects observed by HDX-MS correspond to the average [Δdeuteration] taken from Figures 3 and S6, the scale is from 0 % (blue) to 7.5% (white) to 15 % (red). Grey means no HDX-MS data were obtained.

(1) The interlobe linker corresponds to residues [76-81] and the linker region to residues [74-82].

DATASETS	Holo-CaM	Holo-CaM:CDZ
HDX reaction details		
- *pD:*	7.4	7.4
- *T°C:*	RT	RT
- *Deuterium level*		
• *during labelling*	80%	80%
• *after quench*	26.7%	26.7%
- *Calcium:*	2 mM	2 mM
- *DMSO:*	2%	2%
- *CDZ molar excess* (1)*:*	/	32x
- *% complex during labelling* (2)*:*	/	90
HDX time course analyzed (min)	0.16, 0.33, 0.5, 1, 10, 30, and 120 min	
Number of peptides	40	40
Sequence coverage	93.2%	93.2%
Average peptide length	13.3	13.3
Redundancy	3.85	3.85
Average peptide length / Redundancy ratio	3.45	3.45
Replicates (3)	3	3
Repeatability (pooled SD, Da) (4)	0.06	0.06
Significance difference between states (5)	Wald test, $p<0.05$; biological threshold set to 3%	

S4: **HDX-MS data summary**.

(1) Molar excess used in labelling
(2) K_D = 3 µM; binding stoichiometry Holo-CaM:CDZ =1:1.2
(3) Independent technical replicates
(4) One unique charge state is used per peptide
(5) MEMHDX software (https://memhdx.c3bi.pasteur.fr/)

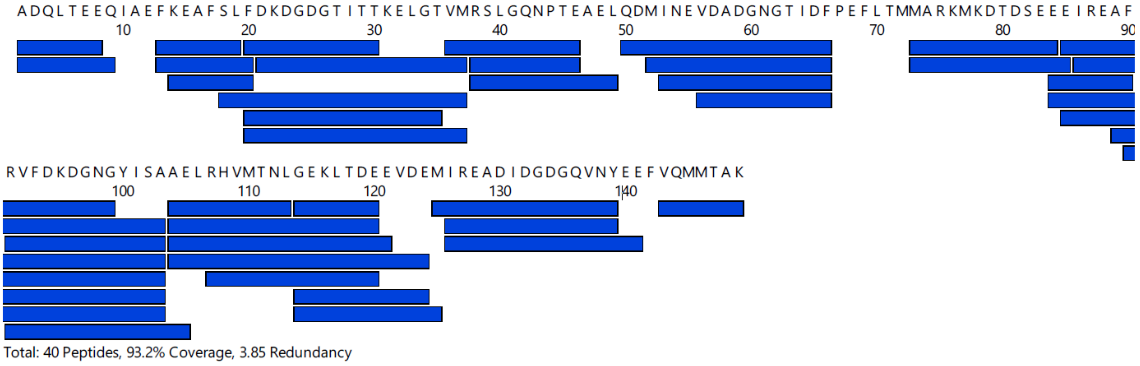

Figure S6: **Peptide map of CaM by HDX-MS.** Peptide map of CaM generated after 2 min pepsin digestion at 20°C and pH 2.5. Each blue bar corresponds to a unique peptide. A total of 40 peptides covering 93.2% of the protein sequence with a 3.85 redundancy value were selected for HDX-MS.

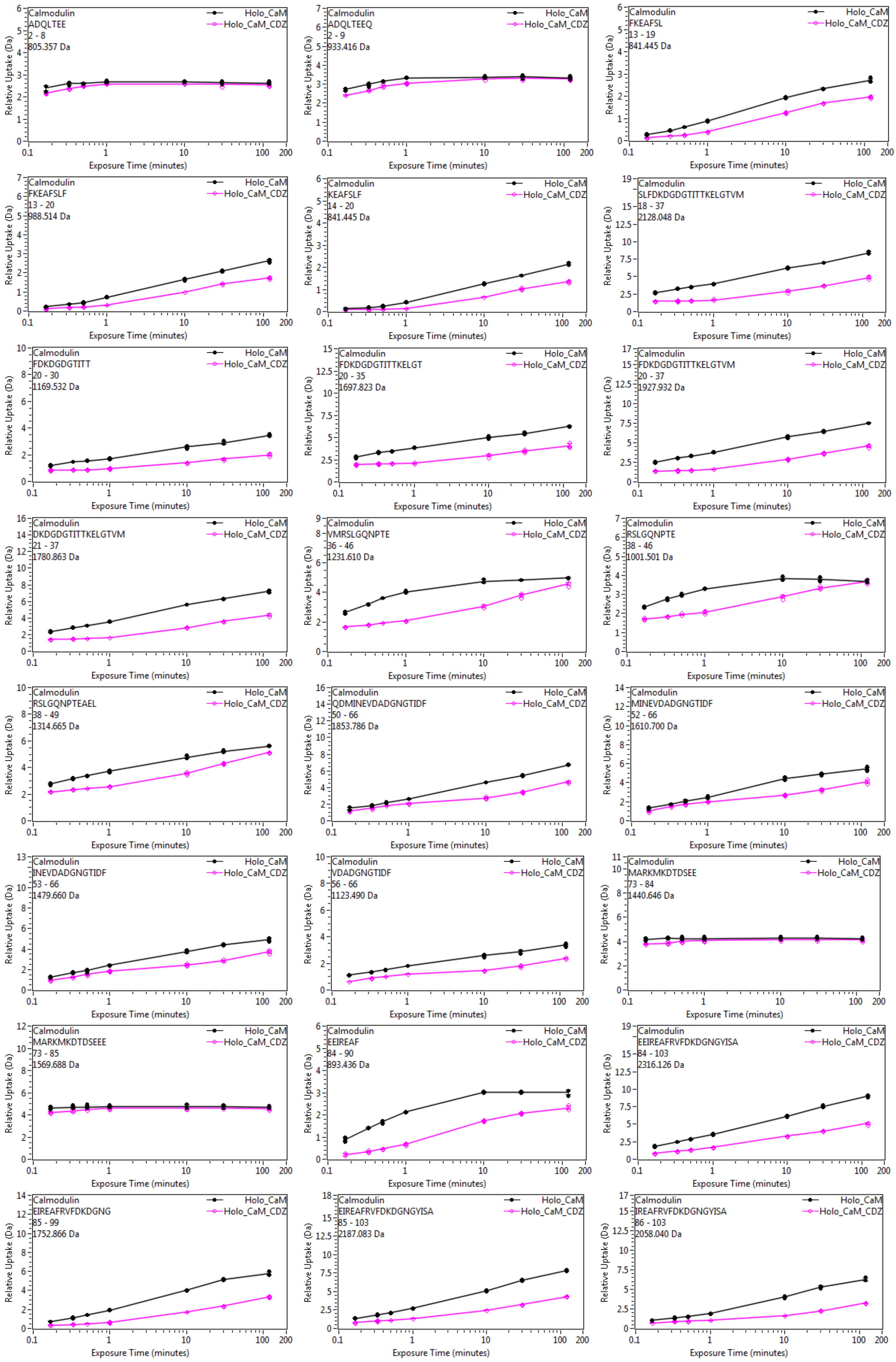

Calmodulin
ADQLTEE
2 - 8
805.357 Da
Calmodulin
ADQLTEEQ
2 - 9
933.416 Da
Calmodulin
FKEAFSL
13 - 19
841.445 Da
Calmodulin
FKEAFSLF
13 - 20
988.514 Da
Calmodulin
KEAFSLF
14 - 20
841.445 Da
Calmodulin
SLFDKDGDGTITTKELGTVM
18 - 37
2128.048 Da
Calmodulin
FDKDGDGTITT
20 - 30
1169.532 Da
Calmodulin
FDKDGDGTITTKELGT
20 - 35
1697.823 Da
Calmodulin
FDKDGDGTITTKELGTVM
20 - 37
1927.932 Da
Calmodulin
DKDGDGTITTKELGTVM
21 - 37
1780.863 Da
Calmodulin
VMRSLGQNPTE
36 - 46
1231.610 Da
Calmodulin
RSLGQNPTE
38 - 46
1001.501 Da
Calmodulin
RSLGQNPTEAEL
38 - 49
1314.665 Da
Calmodulin
QDMINEVDADGNGTIDF
50 - 66
1853.786 Da
Calmodulin
MINEVDADGNGTIDF
52 - 66
1610.700 Da
Calmodulin
INEVDADGNGTIDF
53 - 66
1479.660 Da
Calmodulin
VDADGNGTIDF
56 - 66
1123.490 Da
Calmodulin
MARKMKDTDSEE
73 - 84
1440.646 Da
Calmodulin
MARKMKDTDSEEE
73 - 85
1569.688 Da
Calmodulin
EEIREAF
84 - 90
893.436 Da
Calmodulin
EEIREAFRVFDKDGNGYISA
84 - 103
2316.126 Da
Calmodulin
EIREAFRVFDKDGNG
85 - 99
1752.866 Da
Calmodulin
EIREAFRVFDKDGNGYISA
85 - 103
2187.083 Da
Calmodulin
IREAFRVFDKDGNGYISA
86 - 103
2058.040 Da
Holo_CaM
Holo_CaM_CDZ
Relative Uptake (Da)
Exposure Time (minutes)

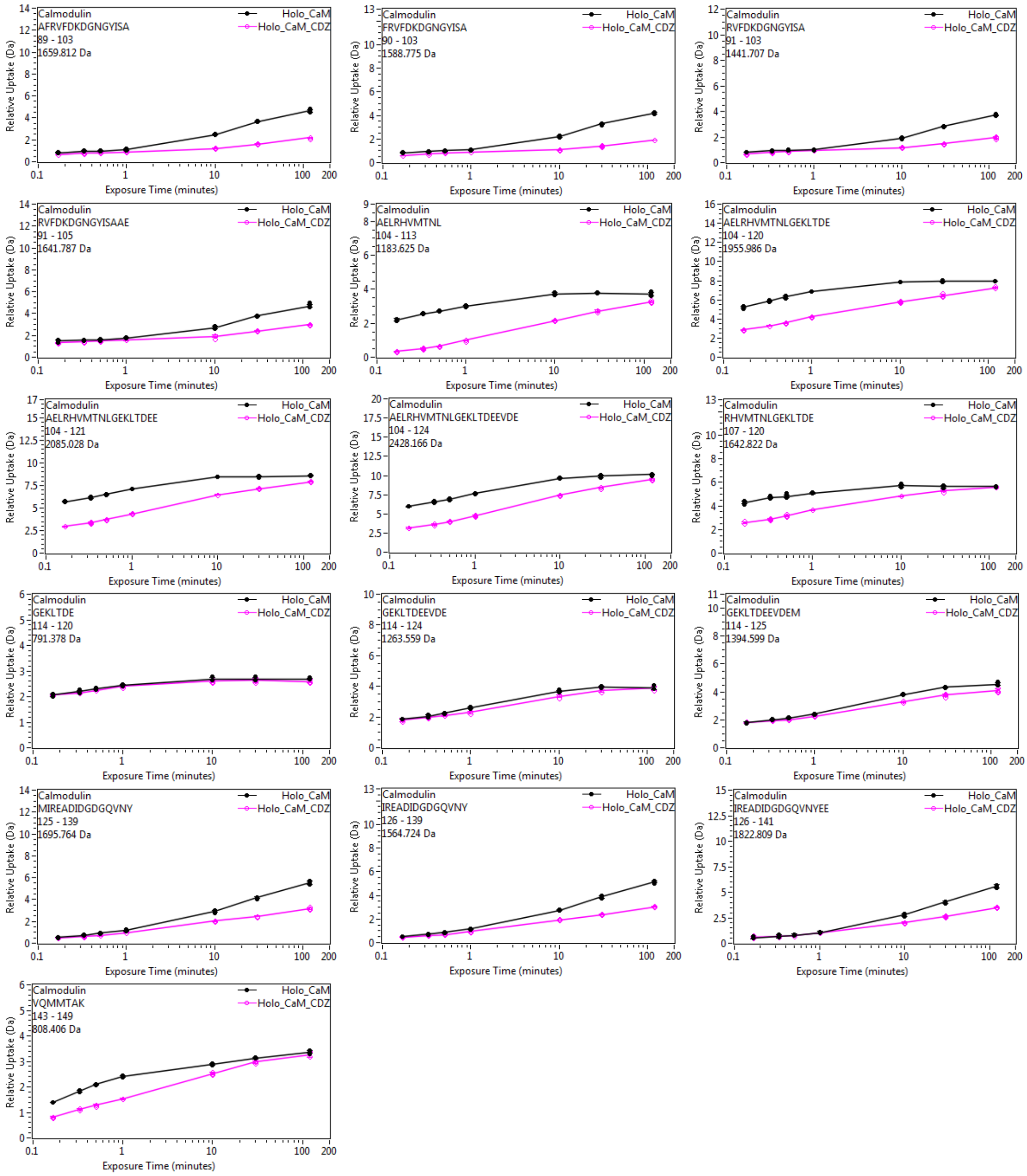

Figure S7: Deuterium uptake plots for each individual CaM peptides across all conditions.

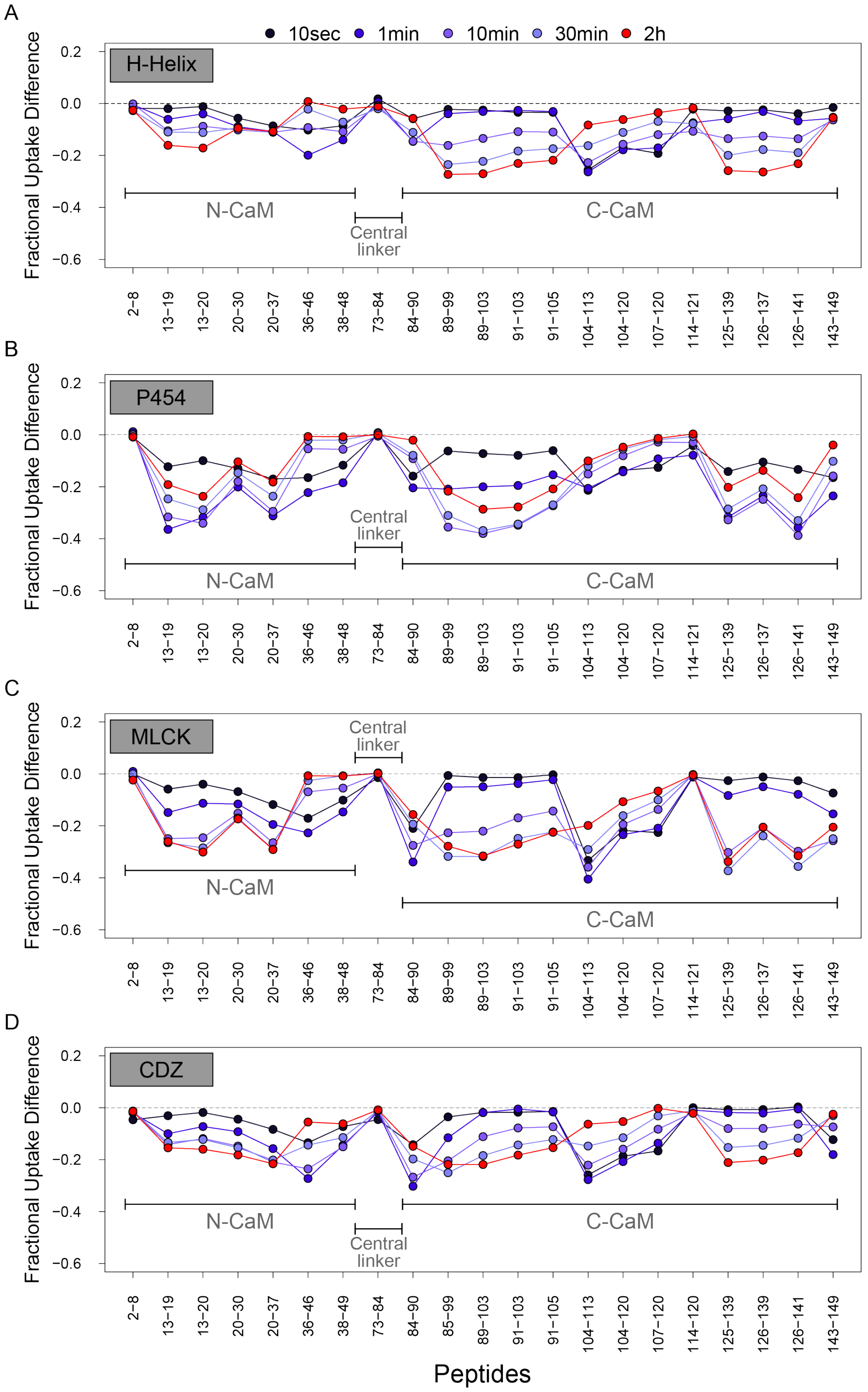

Figure S8: **Comparison of the differential HDX patterns within Holo-CaM upon H-helix (A), P454 (B), MLCK (C) and CDZ (D) binding.** Each differential fractional uptake plot shows the differences in uptake calculated between the CDZ-bound and the free Holo-CaM states at each time point, and for each selected peptide. A negative uptake difference value indicates a ligand-induced protective effect (i.e., reduction in solvent accessibility).

Holo-CaM:CDZ ratio	τ_c (ns) [(1)]
1:0.0	4.5 ± 0.2
1:0.5	4.7 ± 0.2
1:1.0	5.2 ± 0.3
1:2.0	5.4 ± 0.4
1:2.7	5.7 ± 0.1

Table S5: **Rotational correlation time (τ_c) of Holo-CaM at different Holo-CaM:CDZ ratios.** For a protein of a given molecular weight, τ_c is expected to be longer for compact conformations relative to compact domains joined by a flexible linker as is the case of Holo-CaM. The τ_c value increases at a 1:1 Holo-CaM:CDZ ratio (5.2 ± 0.3 ns) relative to Holo-CaM (4.5 ± 0.2 ns) and is effectively the same at a 1:2 ratio (5.4 ± 0.4 ns). Of note, at 0.5 CDZ equivalents, the τ_c (4.7 ± 0.2 ns) corresponds within experimental error to the mean of the free and CDZ-bound protein tumbling times (4.85 ± 0.5 ns), suggesting that free and CDZ-bound (1:1 ratio) forms of Holo-CaM coexist at equivalent concentrations.

(1) τ_c values (± errors) were obtained as described in the Materials and Methods section using the TRACT sequence, which gives tumbling correlation times that are not affected by possible contributions of chemical exchange (between the bound and free forms of a protein or internal conformational exchange) and chemical shift anisotropy.

	Holo-CaM		Holo-CaM : CDZ (1:1)		Holo-CaM : CDZ (1:2)	
	NMR Structure	NMR Shifts	X-Ray	NMR Shifts	X-Ray	NMR Shifts
Residues in Helix	82	84	85	86	85	86
Residues in Strands	4	10	4	12	8	12
Helix (%)	55	57	57	58	57	58
Strand (%)	3	7	3	8	5	8

Table S6: **Comparison of secondary structure content from SRCD, NMR and X-ray.** NMR structure of apo-CaM is a consensus of 25 NMR structures (PDB ID: 1CFC). NMR structure of Holo-CaM is a consensus of three structures of each lobe (PDB ID: 1J7O). NMR and X-ray structures of Holo-CaM with CDZ correspond to the data presented in this paper (PDB ID: 7PSZ, Holo-CaM:CDZ_A 1:1 and PDB ID: 7PU9, Holo-CaM:CDZ_{BC} 1:2).

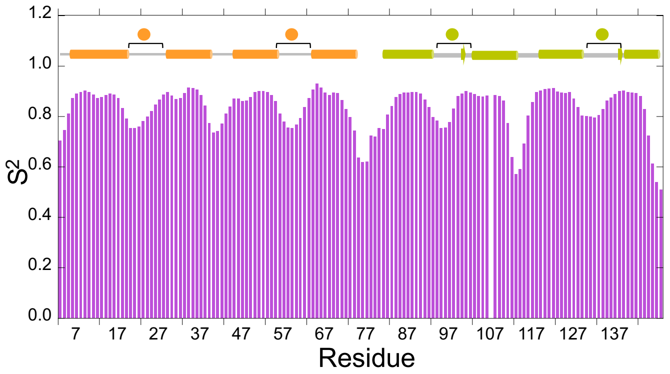

Figure S9. Order parameter (S^2) reflecting the amplitude of motions on the ns-ps time scales of free Holo-CaM. The secondary structure (helix=cylinder, strand=arrow) and calcium ion loops (spheres and square brackets) are schematized on the top of each graph, with the N- and C-lobes colored in orange and green, respectively. Backbone amide S^2 values were calculated from backbone chemical shifts using RCI. The order parameter, which varies from 0 to 1, reflects the amplitude of internal motions of the backbone NH bonds on the ns-ps time scale (0: unrestricted motions; 1: no internal motions). The N and C-termini, calcium binding loops, the loop between helices H6 and H7 and the interlobe linker region (highlighted in grey) show fast high amplitude motions.

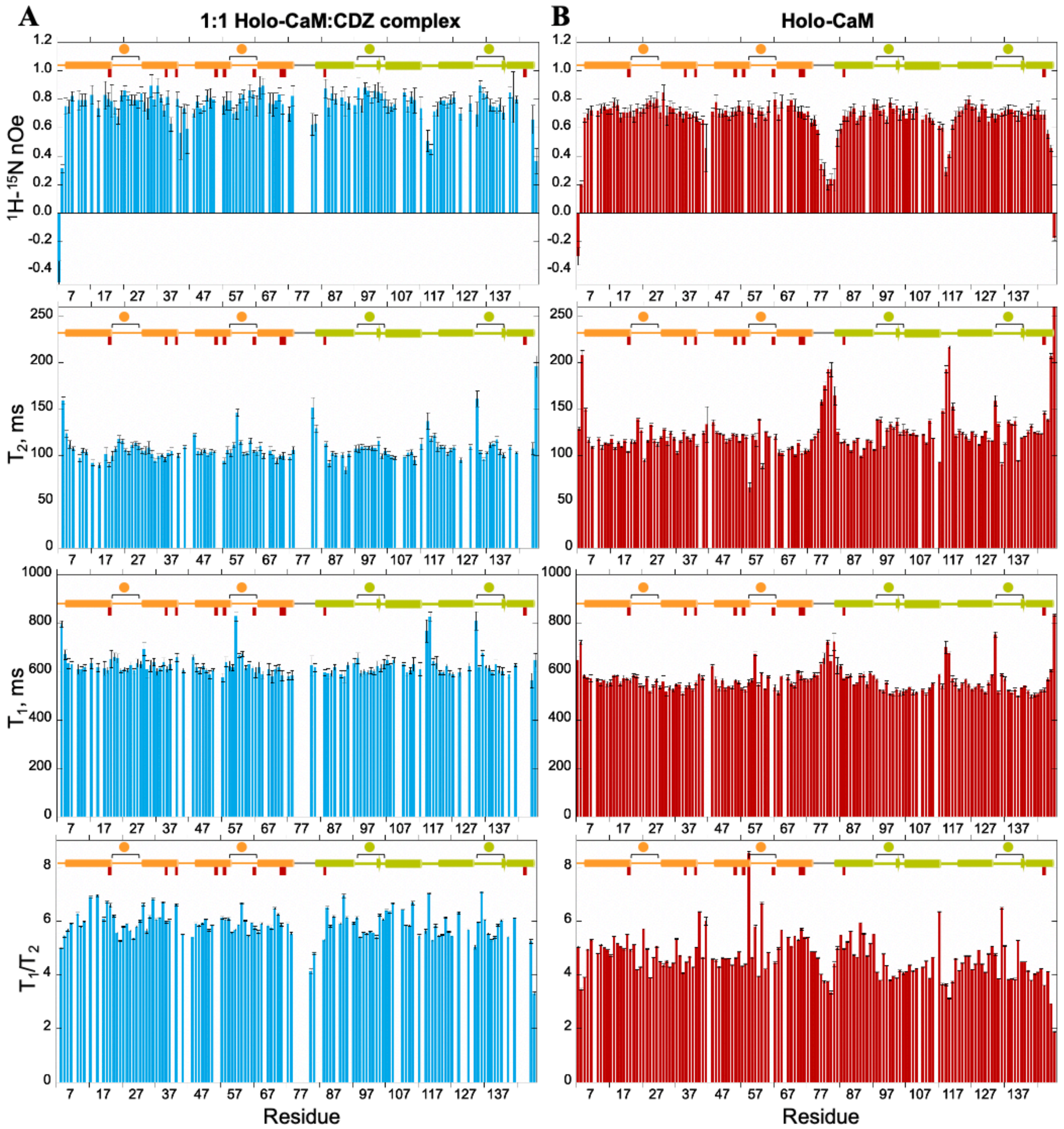

Figure S10: **Holo-CaM internal dynamics from ^{15}N relaxation data.** T_1, T_2 T_1/T_2 and ^{1}H-^{15}N nOe of Holo-CaM in a 1:1 complex with CDZ (**A**) and isolated (**B**). as a function of the residue number. Data were collected at 37 °C on a 600 MHz spectrometer. The secondary structure (helix=cylinder, strand=arrow) and calcium ion loops (spheres and square brackets) are schematized on the top of each graph, with the N- and C-lobes colored in orange and green, respectively. Lacking data are due to either proline residues, signal overlap and unassigned (shifted very weak or absent exchanged-broadened signals). The positions of contacting residues in the X-Ray 1:1 Holo-CaM:CDZ complex structure, as defined by Ligplot+ are represented by wine rectangles. In Holo-CaM, the relaxation parameters (high T_2, low T_1, low T_1/T_2 and low ^{1}H-^{15}N nOes) of residues in the linker region (77-81) and flanking residues in α-helices H4 and H6 are characteristic of high amplitude motions on the ns-ps time scale, with an increased flexibility around its mid-point (79T-80D). In contrast, the relaxation parameters of the α-helical elements of the N- and C-lobes are indicative of ordered regions in a globular protein. The N- (residues 3-4) and C-terminal (146-148) residues are disordered and loops between helices H2-H3 (39-42) and 6-7 (110-118) show increased mobility with respect to the other residues in both lobes' calcium binding EF-hands, as assessed by high T_2 and low ^{1}H-^{15}N nOe values. Finally, residues 55V and 59G in Ca^{2+} binding loop 2, as well as residues 132G and 137N in Ca^{2+} binding loop 4, display low T_2 values revealing conformational exchange phenomena on the ms-μs time scale. Error bars were obtained from 1000 Monte Carlo simulations considering one noise standard deviation.

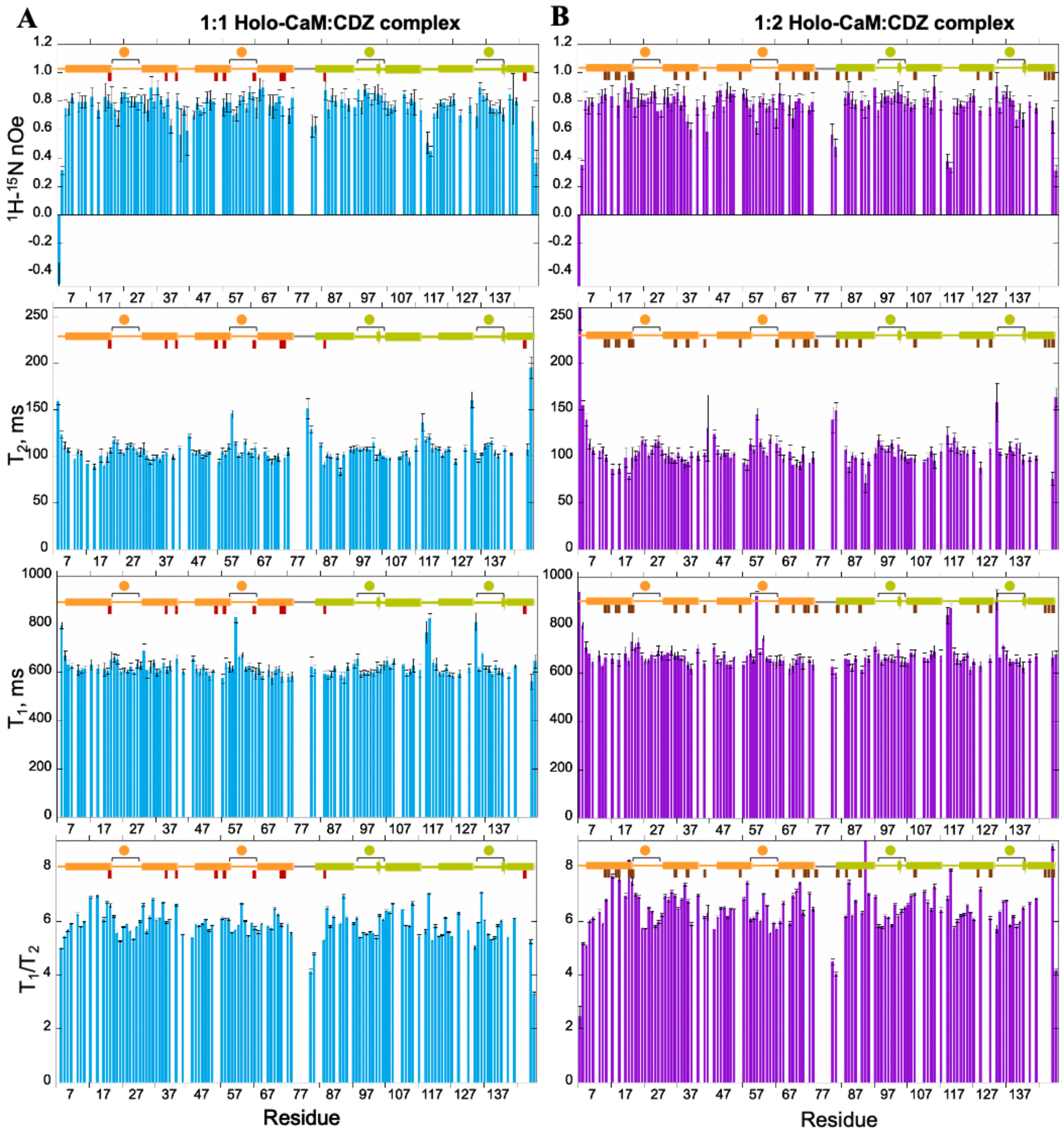

Figure S11: **Comparison of Holo-CaM internal dynamics from ^{15}N relaxation data in complex with CDZ.** T_1, T_2 T_1/T_2 and ^{1}H-^{15}N nOe of Holo-CaM in complex with 1 **A.** or 2 **B.** CDZ molecules as a function of the residue number. The T_1/T_2 ratios, which in the absence of contributions from conformational exchange and for residues in rigid structures (nOe $\geq$ 0.65), are directly related to the tumbling correlation time of a molecule. Data were collected at 37 °C on a 600 MHz spectrometer. Data for the 1:2 complex was obtained with a 3-fold excess of CDZ relative to Holo-CaM. The secondary structure (helix=cylinder, strand=arrow) and calcium ion loops (spheres and square brackets) are schematized on the top of each graph, with the N- and C-lobes colored in orange and green, respectively. Lacking data are due to either proline residues, signal overlap and unassigned (very weak shifted or absent exchanged-broadened signals). The positions of contacting residues in the X-Ray 1:2 Holo-CaM:CDZ complex structure, as defined by Ligplot+ are represented by wine (1:1 complex) and maroon (1:2 complex) rectangles. Error bars were obtained from 1000 Monte Carlo simulations considering one noise standard deviation. The nOe, T_1 and T_2 profiles are very similar in 1 or 2 CDZ-loaded Holo-CaM, albeit some residues showing shorter/longer T_2 values indicative of different conformational exchange rates and slightly higher T_1 values for the 1:3 sample, most likely due to the higher molecular weight of the complex.

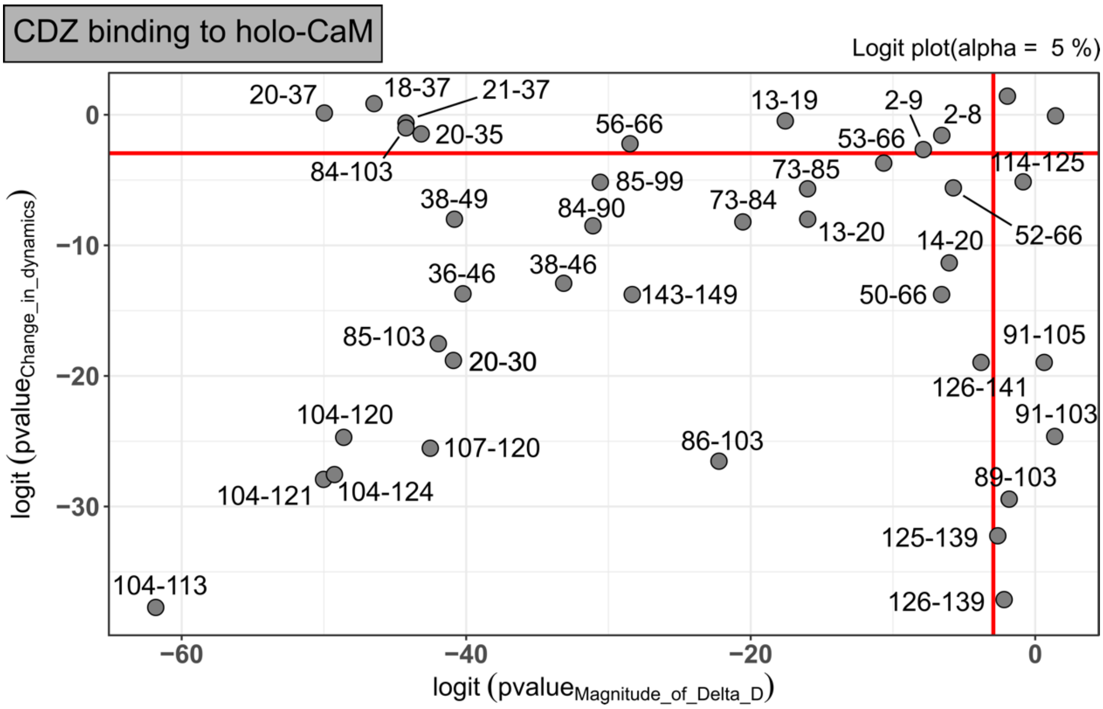

Figure S12: **Statistical analysis performed with MEMHDX**. Logit plot showing the statistical results obtained with all individual CaM peptides in the presence of a 32x molar excess CDZ. The two red lines correspond to the statistically significant threshold used during the statistical analysis (FDR sets to 5%, Wald test, biological threshold sets to 2%). Peptides showing no statistically significant uptake differences between states are displayed on the top right-hand rectangle created by the two red lines. Statistically significant peptides are labelled.
